# Extracellular vesicles from endothelial progenitor cells prevent steroid-induced osteoporosis by suppressing the ferroptotic pathway in mouse osteoblasts based on bioinformatics evidence

**DOI:** 10.1038/s41598-019-52513-x

**Published:** 2019-11-06

**Authors:** Jinsen Lu, Jiazhao Yang, Yongshun Zheng, Xiaoyu Chen, Shiyuan Fang

**Affiliations:** 1 Department of Orthopaedics, Anhui Provincial Hospital, Anhui Medical University, Lujiang Road No. 17, 230001 Hefei, China; 2 Department of Histology and Embryology, Anhui Medical University, Meishan Road No. 81, 230032 Hefei, China

**Keywords:** Mechanisms of disease, Cellular signalling networks

## Abstract

Abnormal antioxidative capabilities were observed in the pathogenesis of steroid-induced osteoporosis (SIOP). Ferroptosis is a recently discovered type of cell death that is characterized by the overproduction of ROS in response to GPX4 and system X^c−^ downregulation, which is mediated by an Fe^2+^ fenton reaction. However, investigations focusing on the relationship between ferroptosis and steroid-induced bone disease remain limited. In the present study, high-dose dexamethasone was used to establish a mouse SIOP model, and extracellular vesicles extracted from bone marrow-derived endothelial progenitor cells (EPC-EVs) alleviated the pathological changes in SIOP via microtomography (micro-CT), with elevations in bone volume (BV), bone surface (BS), trabecular thickness (Tb.Th), and trabecular connectivity density (Conn-D) and decreases in trabecular separation (Tb.sp) and the structure model index (SMI). Histopathological analysis, such as haematoxylin and eosin (HE) and Masson staining, showed that EPC-EVs treatment increased the volume and density of the trabecular bone and bone marrow. RNA sequencing (RNA-seq) and bioinformatics analysis revealed subcellular biological alterations upon steroid and EPC-EVs treatment. Compared with the control, high-dose dexamethasone downregulated GPX4 and system X^C−^, and the Kyoto Encyclopedia of Genes and Genomes (KEGG)-based gene set enrichment analysis suggested that the ferroptotic pathway was activated. In contrast, combination treatment with EPC-EVs partly reversed the KEGG-mapped changes in the ferroptotic pathway at both the gene and mRNA expression levels. In addition, alterations in ferroptotic marker expression, such as SLC3A2, SLC7A11, and GPX4, were further confirmed by RNA-seq. EPC-EVs were able to reverse dexamethasone treatment-induced alterations in cysteine and several oxidative injury markers, such as malondialdehyde (MDA), glutathione (GSH), and glutathione disulphide (GSSG) (as detected by ELISA). In conclusion, EPC-EVs prevented mouse glucocorticoid-induced osteoporosis by suppressing the ferroptotic pathway in osteoblasts, which may provide a basis for novel therapies for SIOP in humans.

## Introduction

Steroids are a large category of anti-inflammatory drugs that are commonly used in inflammation-related syndromes, with potent therapeutic effects. However, the widespread use of these drugs has also caused severe side effects; for example, the prevalence rate of steroid-induced osteoporosis (SIOP) has rapidly increased in recent decades, and the disease incidence has shown a trend towards occurring in younger patients^1,2^. In contrast to the treatment of degenerative osteoporosis, the treatment of SIOP focuses more on drug effects and molecular biological mechanisms, which are closely related to promoting skeletal metabolism and bone remodelling^3,4^. However, calcium supplementation, bisphosphonates, and other classical anti-osteoporosis therapies do not achieve satisfactory results^1^ for patients who relied on steroid drugs. In addition, surgical therapies have been reported to have shortcomings, including severe injury, high complexity, high revision rates and low popularity, owing to the restriction of facilities and the difficulty in obtaining suitable stem cells^5–7^. Therefore, we urgently need to develop simpler and more practical therapies to treat patients with SIOP. Extracellular vesicles are lipid bilayer-enclosed particles that are naturally produced and secreted by cells, which contain various proteins and nucleic acids and mediate cell-to-cell communication to regulate a wide range of biological phenomena, including apoptosis, differentiation, cancer invasion and inhibition, angiogenesis and osteogenesis^8–10^. Among the diverse family of extracellular vesicles, stem cell-derived extracellular vesicles, which function as intercellular messengers among stem cells, were found to play an indispensable role in tissue repair and cell regeneration^11,12^. Various types of extracellular vesicles, including those derived from mesenchymal stem cells (MSCs), mineralizing osteoblasts, monocytes and prostate cancer cells were suggested to be able to accelerate angiogenesis and fracture-healing processes^13,14^. Endothelial progenitor cells (EPCs), which are also referred to as angioblasts, were found to have promising effects on myocardial regeneration in animals. EPC-derived extracellular vesicles (EPC-EVs) have also been reported to have stronger effects on angiogenesis and the healing of cutaneous wounds and are easier to manipulate than EPCs^15^. In addition, due to their specific biological features and structure, rejection and anaphylactic reactions to EPC-EVs have rarely been reported in the literature^16^. Therefore, EPC-EVs were hypothesized to participate in the regeneration of bone and bone-related tissues, and EPC-EVs may be an alternative to classical stem cell implantation for the treatment of SIOP. EPC-EVs repaired traumatic bone defects in animal models, promoting angiogenesis and bone remodelling, but the mechanism needs further investigation^17,18^. Iron metabolism is widely involved in bone growth and production^19,20^. Ferroptosis is a recently discovered type of programmed cell death that is closely related to the disturbance of iron metabolism and is closely related to various bone diseases. Unlike apoptosis, autophagy, and necrosis^21^, the ferroptotic process is characterized by the inhibition of GPX4 and system X_c_^−^ and may lead to the disruption of cysteine metabolism and the enhancement of lipid peroxidation. Finally, an overactive fenton reaction produces a large amount of ROS and leads to cell death^22,23^. To the best of our knowledge, no current studies have reported that high-dose steroid hormones can induce the activation of molecules related to the ferroptotic pathway in certain cell types. The relationship between GPX4 disruption, oxidative stress in osteoblasts, osteoclasts, chondrocytes, and MSCs, suggesting that glucocorticoid administration may be involved in pathogenic processes^24–26^. Therefore, it was hypothesized that steroid hormones may induce ferroptosis or may at least activate the ferroptotic signalling pathway in bone remodelling-related cells, but EPC-EVs may alleviate the symptoms of SIOP, preventing these cells from undergoing death or injury by reversing the activation of the ferroptotic pathway. EPCs and EPC-EVs promoted the angiogenic process, which is essential to bone growth because it provides a sufficient blood supply, but their effects on bone remodelling-related cells, such as osteoblasts, osteoclasts, chondrocytes, and MSCs, are still unclear, and very little big data-based transcriptome and proteome research has been performed on this topic. Therefore, this study focused on exploring the biological effects of EPC-EVs on SIOP in mice in an effort to determine their functional role in altering the whole transcriptomes of osteoblasts to verify the participation of ferroptotic pathways in steroid hormone and EPC-EVs interventions.

## Results

### Isolation and identification of bone marrow EPCs (BM-EPCs)

**Figure 1:**
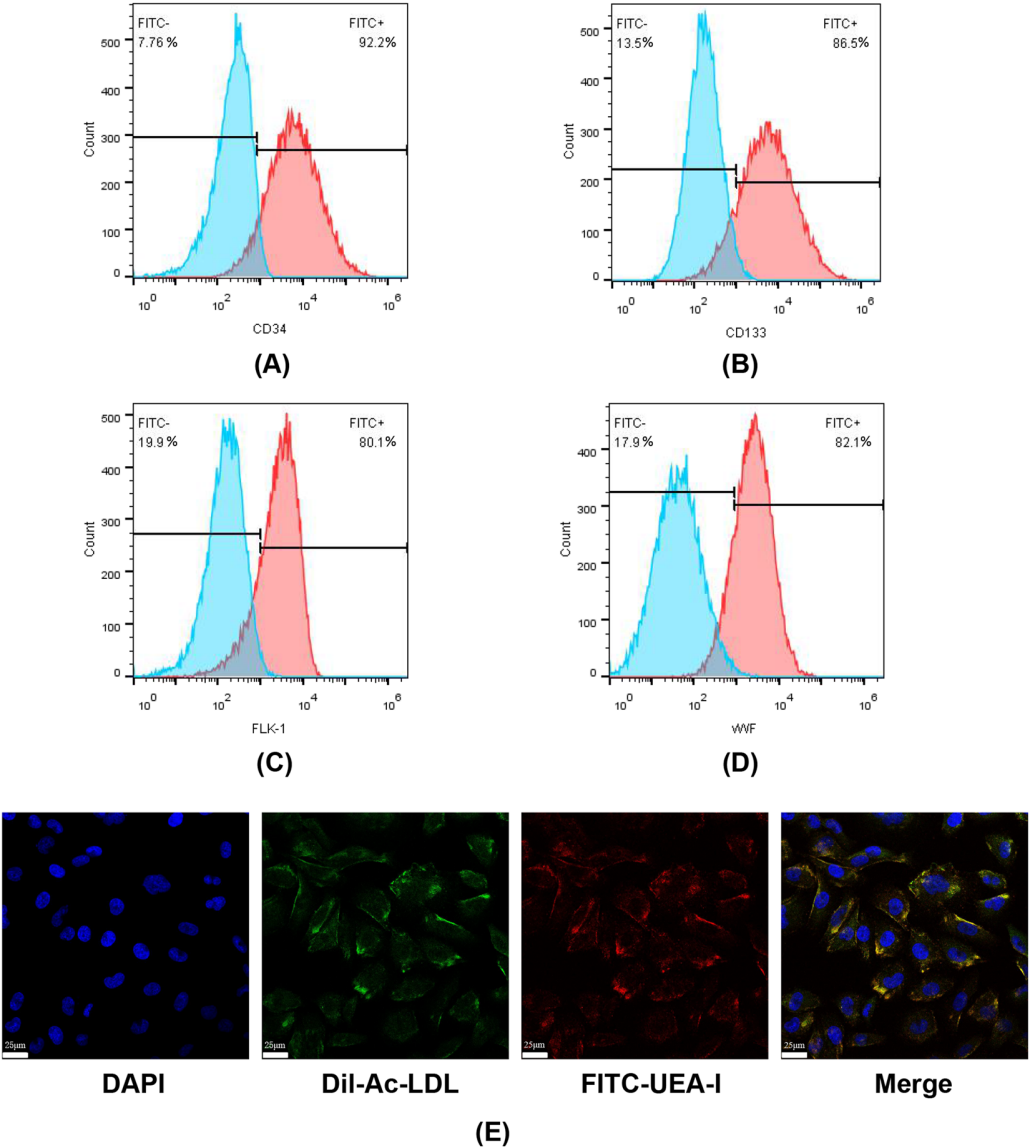
Isolation and characterization of EPCs. BM-EPCs were isolated by density gradient centrifugation and were cultured until they reached the appropriate density. Isolated EPCs were grouped and incubated with FITC-labelled primary antibodies against the surface markers of EPCs, such as CD34, CD133, FLK-1, and vWF. Flow cytometry analysis showed that there were FITC-positive cells with specific EPC surface markers, such as (**A**) CD34, (**B**) CD133, (**C**) FLK-1 and (**D**) vWF. Isolated EPCs that were not incubated with FITC-labelled antibodies were tested as a control. The experimental group is marked in red, and the control group is marked in blue. Both the FITC-positive and FITC-negative cell percentages were calculated and are shown in the image. (**E**) Representative images of the FITC-UEA-I and Dil-Ac-LDL dual-staining of EPCs. Cell nuclei were stained with DAPI (blue fluorescence), FITC-UEA-I is shown in green and Dil-Ac-LDL is shown in red. The merged picture shows the overlay of the results for both FITC-UEA-I and Dil-Ac-LDL staining, showing dual-staining positive cells, which were characterized as EPCs.

EPCs were obtained from mouse bone marrow with density-gradient centrifugation assays, and CD133, CD34, foetal liver kinase 1 (FLK-1) and von Willebrand factor (vWF) were used as surface markers for EPCs, based on the literature^27,28^. To identify the isolated BM-EPCs, cells were cultured until they reached the logarithmic growth phase and were labelled with fluorescein isothiocyanate (FITC)-CD133, FITC-CD34, FITC-FLK-1 and FITC-vWF via immunofluorescence staining. Flow cytometry was utilized to analyse the biomarker expression, such as CD133, CD34, FLK-1 and vWF, on EPCs. Figure 1A–D shows that the proportions of CD133+, CD34+, FLK-1+ and vWF+ cells were 86.5%, 98.2%, 82.1% and 80.0%, respectively. In addition, EPCs were reported to be mostly unipotent stem cells that were able to take up acetylated low-density lipoprotein (Ac-LDL) and to bind to *Ulex europaeus* agglutinin I (UEA-1), leading to neovascularization through either paracrine or autocrine mechanisms^28^. Thus, FITC-UEA-I and 1,1′-dioctadecyl-3,3,3′,3′-tetramethylindocarbocyanine-Ac-LDL (Dil-Ac-LDL) dual-staining were used to identify isolated EPCs, and staining results were detected via confocal laser scanning microscopy. As shown in Fig. 1E, over 90% of cells were double-positive for FITC-UEA-I and Dil-Ac-LDL, indicating that the majority of the cells that we obtained were BM-EPCs, providing the fundamental basis for the following experiments.

### Identification and internalization of EPC-EVs

**Figure 2:**
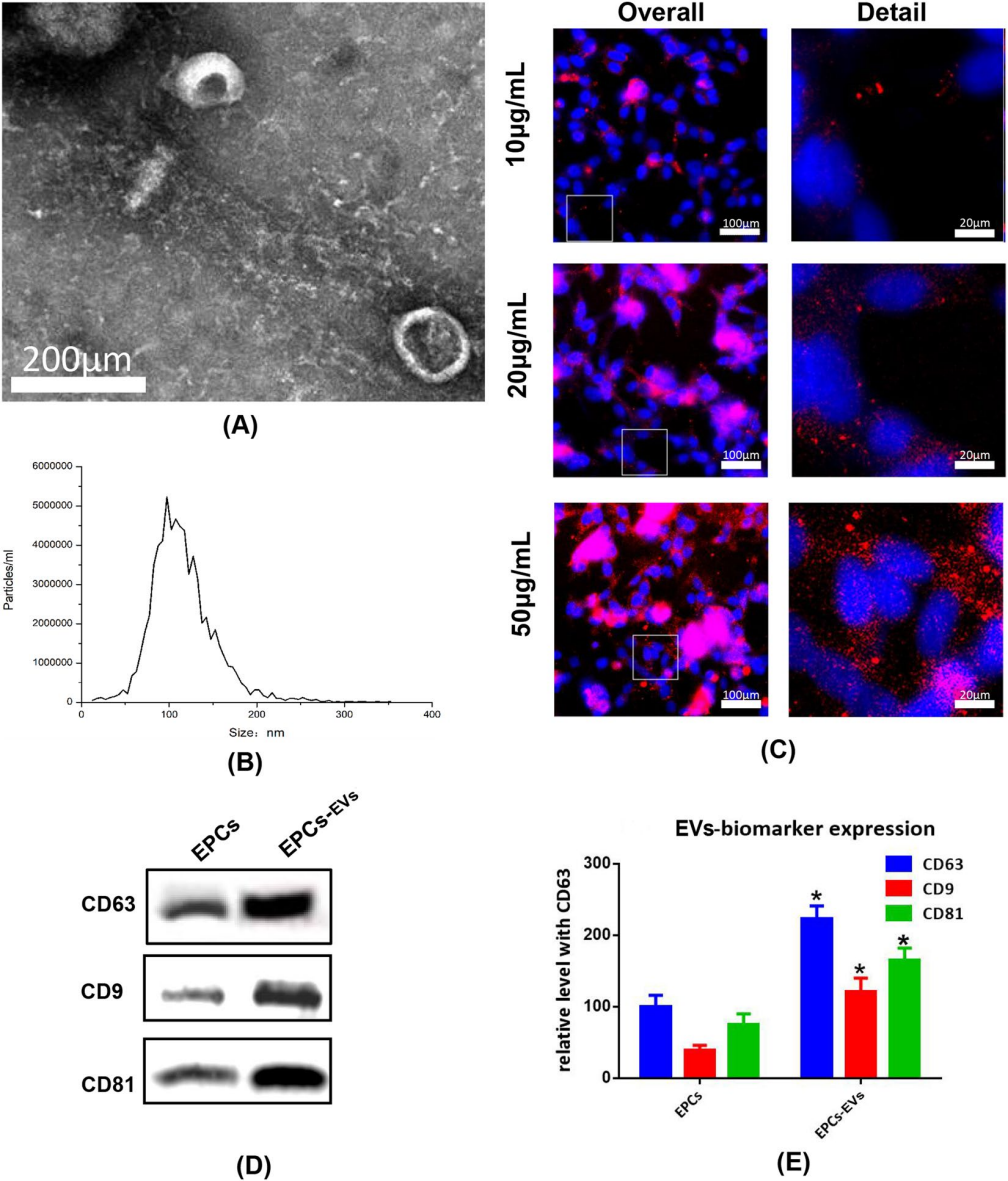
Identification and internalization of EPC-EVs. Extracellular vesicles were isolated from samples with an extracellular vesicle isolation kit from mouse osteoblast medium after specific experimental treatments. (**A**) Morphological features of extracellular vesicles were observed via bio-transmission electron microscopy. (**B**) Particle size of extracellular vesicles was detected with NTA. The X-axis shows the particle size inside of the sample, and the Y-axis shows the concentration of particles with a certain size. Total protein was extracted from extracellular vesicles and analysed with western blotting. Representative images (**D**,**E**) histograms showing the expression levels of CD9, CD63, and CD81, which are surface markers of extracellular vesicles. PKH26-labelled extracellular vesicles at 10, 20, 50 µg/mL (approximately 0.69 × 10^10^, 1.38 × 10^10^ and 3.45 × 10^10^ vesicles) were co-cultured with mouse osteoblasts for 3 hours. (**C**) Immunofluorescence images show the uptake of extracellular vesicles by osteoblasts. The nucleus was labelled with DAPI (blue), and PKH26-labelled extracellular vesicles were internalized by osteoblasts (red).

Mouse bone marrow-derived EPC-EVs were isolated with an extracellular vesicle extraction kit and were identified based on the particle size, surface markers, and morphological features. Initially, isolated extracellular vesicles were detected with a transmission electron microscope to examine the morphological features, and the observed 80–120 nm disc-like structures had characteristics consistent with extracellular vesicles (Fig. 2A). Next, nanoparticle tracking analysis (NTA) was conducted to analyse the concentration and particle-size distribution of the extracellular vesicles. As shown in Fig. 2B, the particle sizes mostly ranged from 80–140 nm, indicating that these extracellular vesicles were high quality. To further examine the biological features of the extracellular vesicles, isolated extracellular vesicles were lysed, the typical extracellular vesicle biomarkers, such as CD9, CD63 and CD81, were evaluated by western blotting, and the density of each band was normalized to the total protein. As shown in Fig. 2D,E, with equal loading conditions, the quantity of CD9, CD63 and CD81 was clearly higher in EPC-EVs than in the EPC control. Since the effective absorption of extracellular vesicles into mouse osteoblasts was one of the prerequisites for further treatment experiments, the extracellular vesicle uptake ability of mouse osteoblasts was tested with fluorescence microscopy after an incubation with PKH26-labelled extracellular vesicles. These results showed that the number of PKH26-labelled fluorescent spots gradually increased inside the osteoblasts, which indicated that osteoblasts could effectively internalize extracellular vesicles in a dose-dependent manner (Fig. 2C).

### Microtomography (micro-CT) analysis of EPC-EVs in an SIOP mouse model

**Figure 3:**
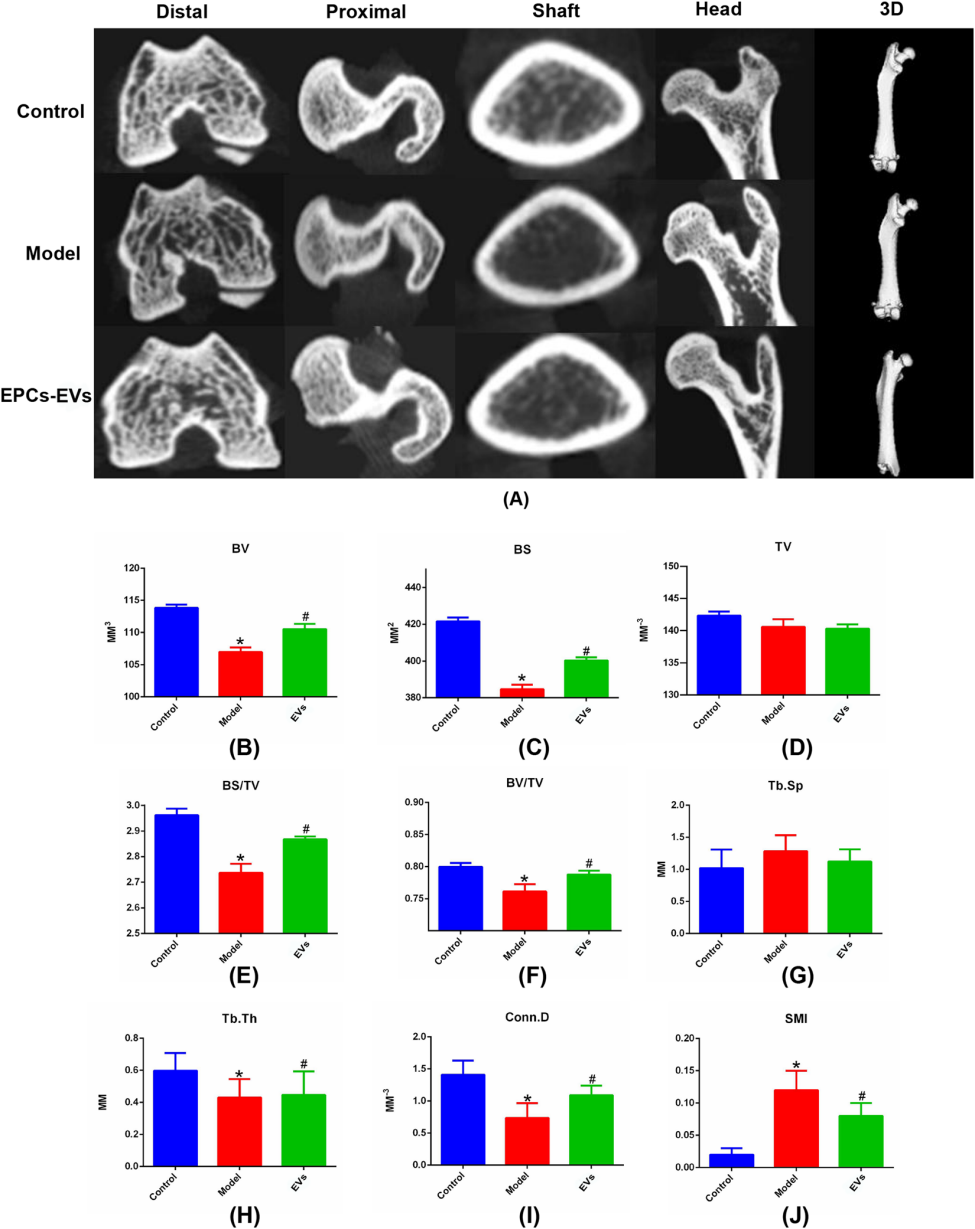
Micro-CT analysis of femurs in SIOP mice treated with EPC-EVs. After animals were grouped and treated, mouse femurs were recovered and fixed for micro-CT scanning. (**A**) 2-D images of the coronal plane and the cross-section show the microstructure of the femurs from various perspectives, including the distal femur, proximal femur, femur shaft and femur head. 3-D reconstruction images show the overall shape of the femurs. (**B**) Osteoporosis-related parameters were analysed with software based on micro-CT scanning data, such as TV, BS, BV, BS/TV, BV/TV, BS/BV, Conn-D, Tb.sp, Tb.Th and the SMI. *P < 0.05 vs control and ^#^p < 0.05 vs the model group were considered statistically significant.

A SIOP mouse model was generated by the intramuscular administration of 20 mg/kg dexamethasone. After the establishment of the SIOP model, 50 µg of EPC-EVs (approximately 3.45 × 10^10^ EPC-EVs) were introduced into the mouse circulation via tail vein injections once a week for 4 weeks, and all mice were sacrificed 2 weeks after the treatment. After sacrifice, the mouse femurs from the respective groups were fixed and scanned by micro-CT. Sectional coronal and sagittal images and cross-sections were collected and analysed based on the scanning results of micro-CT. The 2-D images were converted into 3-D images to show the overall changes in the femur samples. The 2-D images showed the microstructure of diverse parts of the femurs, including the distal femur, proximal femur, femur shaft, and femur head. Compared with the control group, the SIOP model group had reduced density of the cancellous bone and the trabecular bone at the proximal and distal ends of the femur. In addition, a slightly thinned cortex of the femoral shaft and a larger medullary cavity were observed in the SIOP model group. In addition, a section of the femoral head showed collapsed cartilage and increased bone density, indicating the occurrence of necrosis in the SIOP group. Compared with the model group, the EPC-EV treatment group had higher cancellous bone and trabecular bone density at the proximal and distal ends of the femur, a slightly thicker femoral shaft cortex, and a smaller medullary cavity. Additionally, the morphological changes in the femoral head cartilage that were observed in the pathological condition were also reversed by the EPC-EV treatment (Fig. 3A). For further quantitative analysis, osteoporosis-related bone parameters, such as tissue volume (TV), bone surface (BS), bone volume (BV), BS/TV, BV/TV, BS/BV, trabecular connectivity density (Conn-D), trabecular separation (Tb.sp), trabecular thickness (Tb.Th) and the structure model index (SMI) were analysed based on the data from micro-CT. Compared with the control group, the SIOP model group had remarkably decreased BS, BV, BS/TV, BV/TV, Tb.Th, and Conn-D and an elevated SMI and Tb.Sp, indicating that the SIOP model had been successfully established. After EPC-EV treatment, BS, BV, BS/TV, BV/TV, Tb.Th, and Conn-D had evidently recovered, and the SMI and Tb.Sp had decreased, indicating that EPC-EVs may have potential as a treatment for SIOP (Fig. 3B–J).

### Histopathological analysis of EPC-EVs in an SIOP mouse model

**Figure 4:**
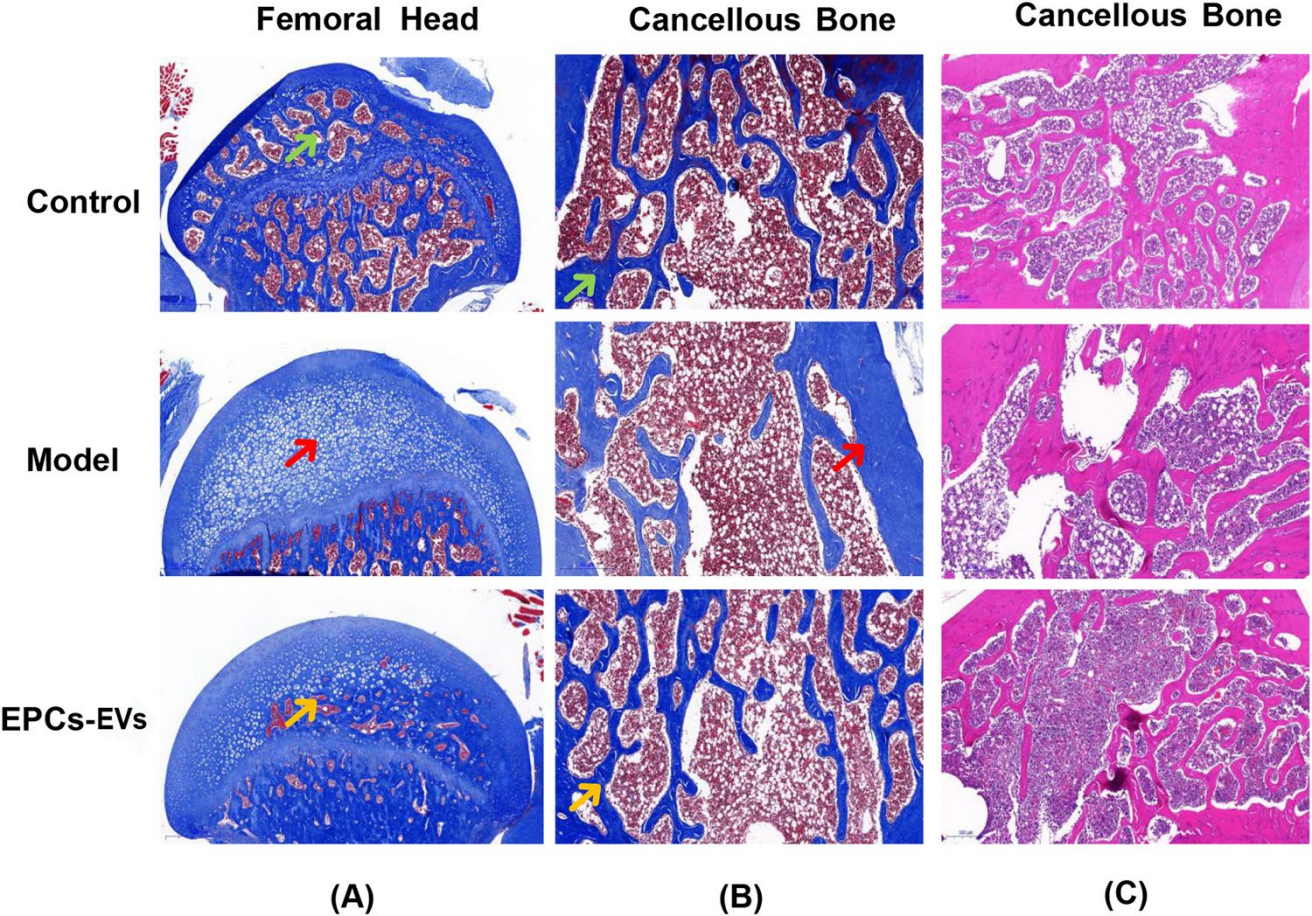
Histopathological staining of femurs in mice with SIOP following EPC-EV treatment. After the treatment of each group, all animals were sacrificed, and their bilateral femurs were recovered, fixed, and decalcified, histopathological sections were prepared for staining. (**A**) Representative pictures of Masson staining in the coronal plane of the femoral head from each group. The green arrow shows the normal structure of the femoral head. The red arrow shows the necrotic tissue that replaced the normal bone structure and marrow. The yellow arrow shows the partially reduced necrosis and recovered blood supply. (**B**) Representative pictures of Masson staining in the cancellous bone of femurs from each group. The green arrow shows normal collagen staining following Masson staining. The red arrow shows that there was less abundant collagen with a light blue colour after Masson staining. The yellow arrow shows the partial recovery of collagen abundance. (**C**) Representative pictures of HE staining in a coronal plane of the cancellous bone from each group.

Histopathological staining was further evaluated to examine the therapeutic effects of EPC-EVs in animals. After the SIOP model generation and EPC-EV treatment, mouse femurs from the various groups were fixed, decalcified and stained, which included Masson and haematoxylin and eosin (HE) staining. Compared with that in the control group, the collagen in the SIOP model group exhibited lightly coloured and sparsely arranged Masson staining. In addition, HE staining showed that the trabecular bone was sparse and reduced in the SIOP model group compared to the control group. In the EPC-EV treatment group, the collagen in the Masson staining was deeply and uniformly stained, and HE staining showed an increase in the amount of trabecular bone and a decrease in the trabecular space, indicating that EPC-EVs alleviated SIOP (Fig. 4B,C). In addition, these results indicated that compared with the control group, the model group had necrotic tissue that replaced the normal bone marrow and the trabecular bone in the femoral head. After EPC-EV treatment, the volume and density of the trabecular bone and bone marrow increased, and the osteonecrosis was reduced in the femoral head, indicating that EPC-EVs may be used as a potential therapy for the steroid-induced necrosis of the femoral head (Fig. 4A).

### Heatmap and volcano plot of differentially expressed genes (DEGs) following EPC-EV treatment in mouse osteoblasts after dexamethasone induction

**Figure 5:**
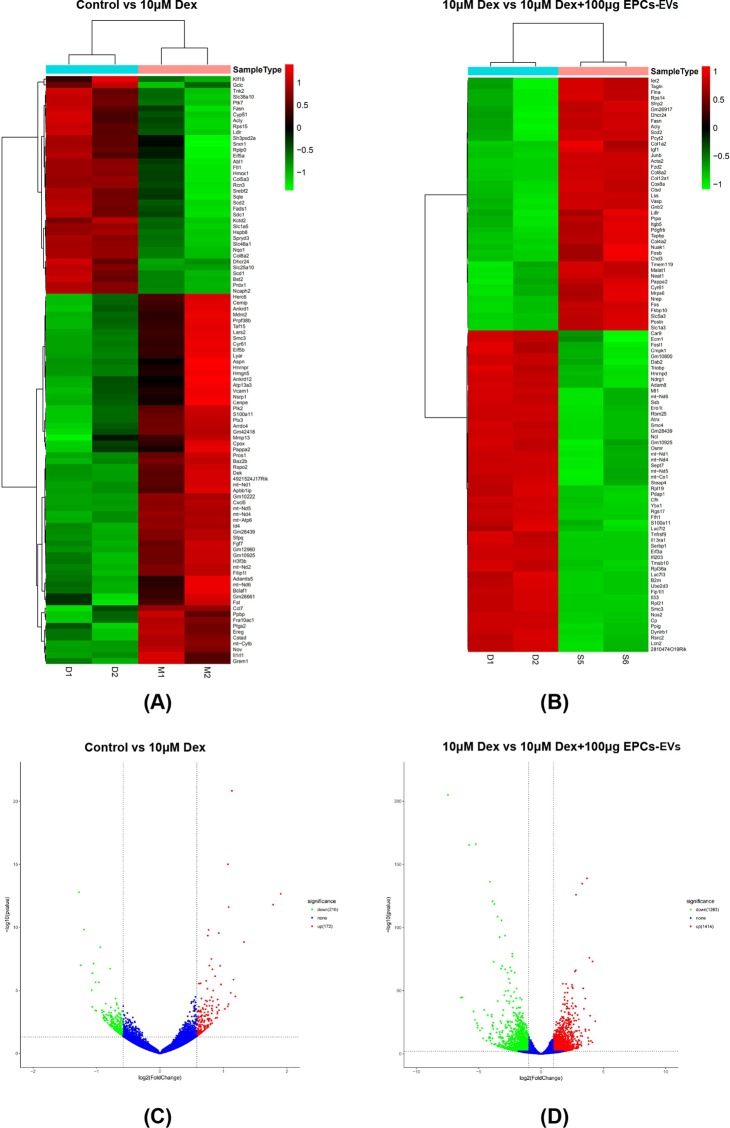
Bioinformatics analysis of the differential gene expression. Total RNA was extracted from osteoblasts from mice that received 10 μM dexamethasone and 10 µM dexamethasone combined with 100 µg EPC-EVs. RNA-seq was conducted, and bioinformatics analysis was performed. (**A**,**B**) Pie chart results showing the read mapping data from two biological replicates from the dexamethasone treatment group in the exons, introns and other categories. (**C**,**D**) Pie chart results show the read mapping information from 2 biological replicates from the dexamethasone + EPC-EV treatment group in the exons, introns and intergenic areas. (**E**) Heat map of the top 100 DEGs between osteoblasts in the dexamethasone and dexamethasone + EPC-EV treatmentgroups. Red indicates higher expression, and green indicates lower expression. (**F**) Volcano plot of DEGs between osteoblasts in the dexamethasone and the dexamethasone + EPC-EV treatment groups. Genes with significantly different expression are represented by red dots (upregulated) and green dots (downregulated). Genes without significantly different expression are represented by blue dots. The abscissas represent the fold change in genes in the two experimental groups, and the ordinates represent the statistical significance of the gene expression changes.

Mouse osteoblasts were cultured until they reached the logarithmic growth phase and were divided into three groups, a normal control group, a group that received 10 μM dexamethasone and a group that received 10 μM dexamethasone plus 100 μg (approximately 6.9 × 10^10^ EPC-EVs) of EPC-EVs for 72 hours. Total RNA was extracted from the three groups, two biological replicates were set up, and RNA sequencing (RNA-seq) was conducted after performing quality control. After sequencing, gene expression level analysis was performed, and then DEG analysis was performed using the read count data that was obtained from the gene expression level analysis. Volcano plots were generated to visually represent the distribution of DEGs. As shown in this figure, DEGs are represented by red dots (upregulated) and green dots (downregulated). Non-DEGs are represented by blue dots. The abscissas represent the fold change in genes between the two experimental groups, and the ordinates represent the significance in the fold change in the gene expression. A total of 2677 DEGs, which included 1414 upregulated and 1263 downregulated DEGs, were identified in osteoblasts from mice that received 10 μM dexamethasone and 10 μM dexamethasone with 50 μg of EPC-EVs (approximately 3.45 × 10^10^ EPC-EVs) (Fig. 5C). In addition, a total of 2677 DEGs, which included 172 upregulated and 216 downregulated DEGs, were identified in osteoblasts from mice in the normal control and 10 μM dexamethasone groups (Fig. 5D). Genes with the same or similar expression patterns were clustered and visualized by heat maps. The linkage in the dendrograms adjacent to the heatmap shows the functional similarity or sharing of the same metabolic and signalling pathways between two genes. Red represents higher expression, and green represents lower expression (Fig. 5A,B).

#### Gene Ontology (GO) and Kyoto Encyclopedia of Genes and Genomes (KEGG) enrichment analysis of DEGs following EPC-EV treatment in mouse osteoblasts after dexamethasone induction

Osteoblasts were pre-treated with 10 µM dexamethasone and a combination of 10 µM dexamethasone and 100 µg (approximately 6.9 × 10^10^ EPC-EVs) of EPC-EVs for 72 hours. Total RNA was extracted and analysed with RNA sequencing. The DEGs were mapped onto specific pathways in 3 categories in the GO database, which included molecular function, cellular component and biological process, by gene set enrichment analysis. For each category, the top 10 pathways are listed. Based on our analysis, pathways including DNA-templated transcription, DNA-templated regulation of transcription and positive regulation of transcription from the RNA polymerase II promoter were associated with the most DEGs in the biological process category. In addition, pathways including the nucleus, cytoplasm, and membrane were associated with more DEGs in the cell component category. Finally, more DEGs were enriched in pathways including protein binding, metal ion binding, and nucleotide binding in the molecular function category (Fig. S2). DEGs were also mapped to the KEGG database, which includes a series of well-organized maps of known biological pathways and explicitly labels each proven molecular-level participant in these maps based on genome sequencing or other high-throughput experimental technologies. This analysis showed that the top 10 pathways are Metabolic pathways, Ribosome, Pathways in cancer, Proteoglycans in cancer, the Thyroid hormone signalling pathway, Influenza A, the Chemokine signalling pathway, the PI3K-Akt signalling pathway, Steroid biosynthesis and Cytokine-cytokine receptor interaction (Fig. S3).

#### DEG enrichment analysis of ferroptotic pathway members in osteoblasts from mice that received dexamethasone and EPC-EV treatment with the KEGG database

**Figure 6:**
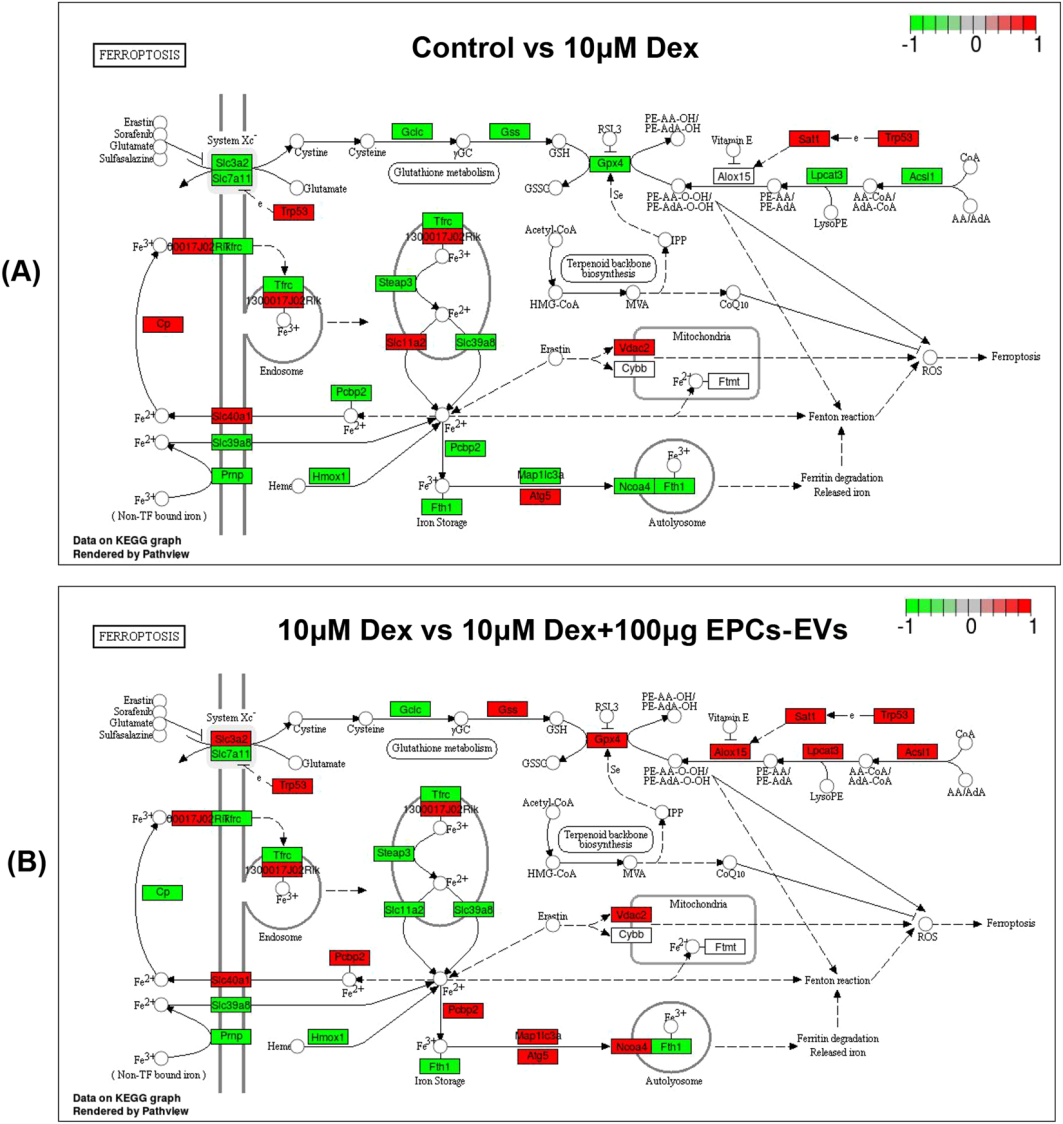
Ferroptotic pathway gene enrichment analysis based on the KEGG database. DEGs were labeled into the map of ferroptosis pathway obtained from KEGG database with official permission and guidance^57–59^. (**A**) DEGs between the control and 10 µM dexamethasone treatment groups were enriched in the KEGG ferroptotic pathway based on 2 biological replicates. The downregulated genes are labelled with green, and the upregulated genes are labelled with red. (**B**) DEGs between 10 µM dexamethasone and the 10 µM dexamethasone + 100 µg EPC-EV treatment groups were enriched in the KEGG ferroptotic pathway based on 2 biological replicates. The downregulated genes are labelled with green, and the upregulated gene are labelled with red.

To examine whether high-dose dexamethasone treatment activated the ferroptotic pathway, gene enrichment analysis was conducted that concentrated on the DEGs in the ferroptotic pathway between mouse osteoblasts from the normal group and from the 10 µM dexamethasone induction group that mapped to the KEGG database. The ferroptotic enrichment analysis demonstrated that Gpx4, Slc3a2, Slc7a11, Trp53, Tfrc, Steap3, Slc11a2, Pcbp2, Slc40a1, Cp, Slc39a8, Prnp, Hmox1, Fth1, Map1lc3a, Atg5, Lpcat3 and Acsl1 were differentially expressed; upregulated genes are labelled in red, and downregulated genes are labelled in green (Fig. 6A). As shown in the labelled pathway map, Gpx4 and system Xc-, two key mediators of ferroptosis, were downregulated, indicating that glutamate and cysteine metabolism might have been disrupted and that more ROS might have been produced, thus triggering ferroptosis following high-dose dexamethasone treatment. Since high-dose dexamethasone was found to activate the ferroptotic pathway based on RNA-seq analysis, we further examined whether EPC-EVs could affect the dexamethasone-induced ferroptotic changes in mouse osteoblasts based on RNA-seq. KEGG pathway enrichment analysis was conducted that focused on the DEGs in the ferroptotic pathway between osteoblasts from mice that received dexamethasone induction alone or combined with 100 µg (approximately 6.9 × 10^10^ EPC-EVs) of EPC-EV treatment that mapped to the KEGG database. These results showed that Gpx4, Slc3a2, Slc7a11, Trp53, Tfrc, Steap3, Slc11a2, Pcbp2, Slc40a1, Cp, Slc39a8, Prnp, Hmox1, Fth1, Map1lc3a, Atg5, Lpcat3 and Acsl1 were differentially expressed (Fig. 6B). As shown in the labelled pathway map, Gpx4 and system Xc- were two key mediators. However, compared with the aforementioned enrichment results, the upregulated and downregulated DEGs between the two experimental groups in the ferroptotic pathway that were mapped in the KEGG database were significantly different, as some DEGs were reversed following EPC-EV treatment. Key mediators, such as Gpx4 and system Xc, were upregulated, indicating that glutamate and cysteine metabolism may have recovered in response to EPC-EVs and that fewer ROS might have been produced, thus triggering less ferroptosis.

### Differentially expressed mRNA enrichment analysis with the KEGG database of the ferroptotic pathway in osteoblasts from mice that received dexamethasone and EPC-EV treatment

**Figure 7:**
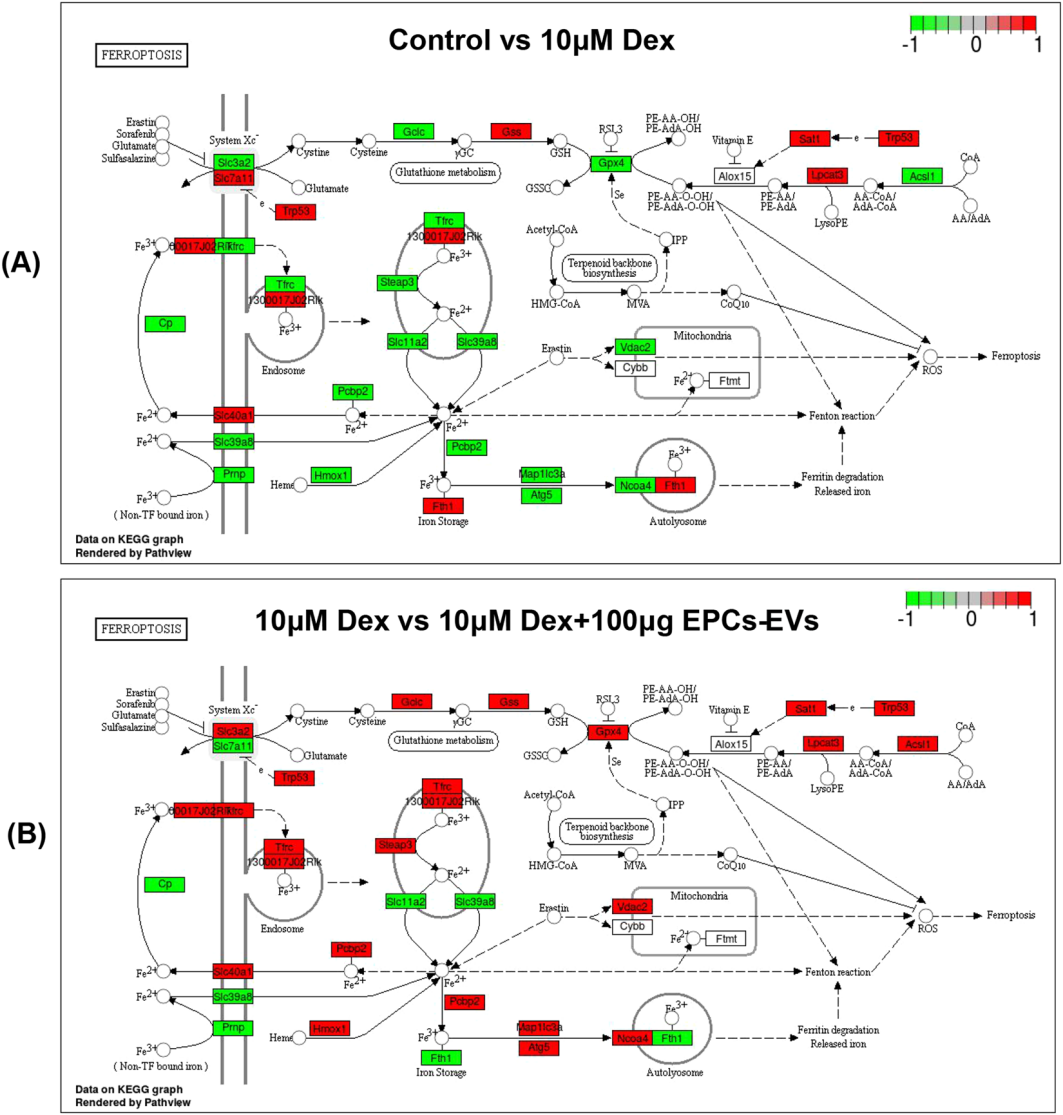
Ferroptotic pathway mRNA enrichment analysis based on the KEGG database. Differentially expressed mRNA were labeled into the map of ferroptosis pathway obtained from KEGG database with official permission and guidance^57–59^. (**A**) Differentially expressed mRNA between the control and 10 µM dexamethasone treatment groups in the KEGG ferroptotic pathway based on 2 biological replicates. The downregulated genes are labelled with green, and the upregulated genes are labelled with red. (**B**) Differentially expressed mRNA between the 10 µM dexamethasone and 10 µM dexamethasone + 100 µg EPC-EV treatment groups in the KEGG ferroptotic pathway based on 2 biological replicates. The downregulated genes are labelled with green, and the upregulated genes are labelled with red.

Apart from DEG-based analyses, mRNA enrichment analysis that concentrated on the differentially expressed mRNA in the ferroptotic pathway was also performed with the KEGG database, examining mRNA in osteoblasts from mice in the normal group and in mice that received 10 µM dexamethasone induction. These results indicated that Gpx4, Slc3a2, Slc7a11, Trp53, Tfrc, Steap3, Slc11a2, Pcbp2, Slc40a1, Cp, Slc39a8, Prnp, Hmox1, Fth1, Map1lc3a, Atg5, Lpcat3 and Acsl1 mRNA were differentially expressed (Fig. 7A). As shown in the labelled pathway map (red represents elevated mRNA, and green represents reduced mRNA), Gpx4 and system Xc- mRNA were downregulated. In addition, KEGG pathway enrichment analysis was also conducted, focusing on differentially expressed mRNA in the ferroptotic pathway for genes that mapped to the KEGG database, examining mRNA in osteoblasts from mice that received dexamethasone induction alone or in combination with 50 µg EPC-EV treatment. These results showed that Gpx4, Slc3a2, Slc7a11, Trp53, Tfrc, Steap3, Slc11a2, Pcbp2, Slc40a1, Cp, Slc39a8, Prnp, Hmox1, Fth1, Map1lc3a, Atg5, Lpcat3, and Acsl1 mRNA were differentially expressed, and Gpx4 and system Xc- mRNA were upregulated, which was consistent with the DEG analysis (Fig. 7B). Briefly, high-dose dexamethasone treatment may activate the ferroptotic pathway by downregulating Gpx4 and System Xc-, and EPC-EVs counteract the activation of the ferroptotic pathway by recovering Gpx4 and System Xc- levels, which may partly explain the therapeutic effects of EPC-EVs in SIOP animal models.

### Confirmation of the regulated patterns in the ferroptotic pathway in osteoblasts from mice that received dexamethasone or EPC-EV treatment

**Figure 8:**
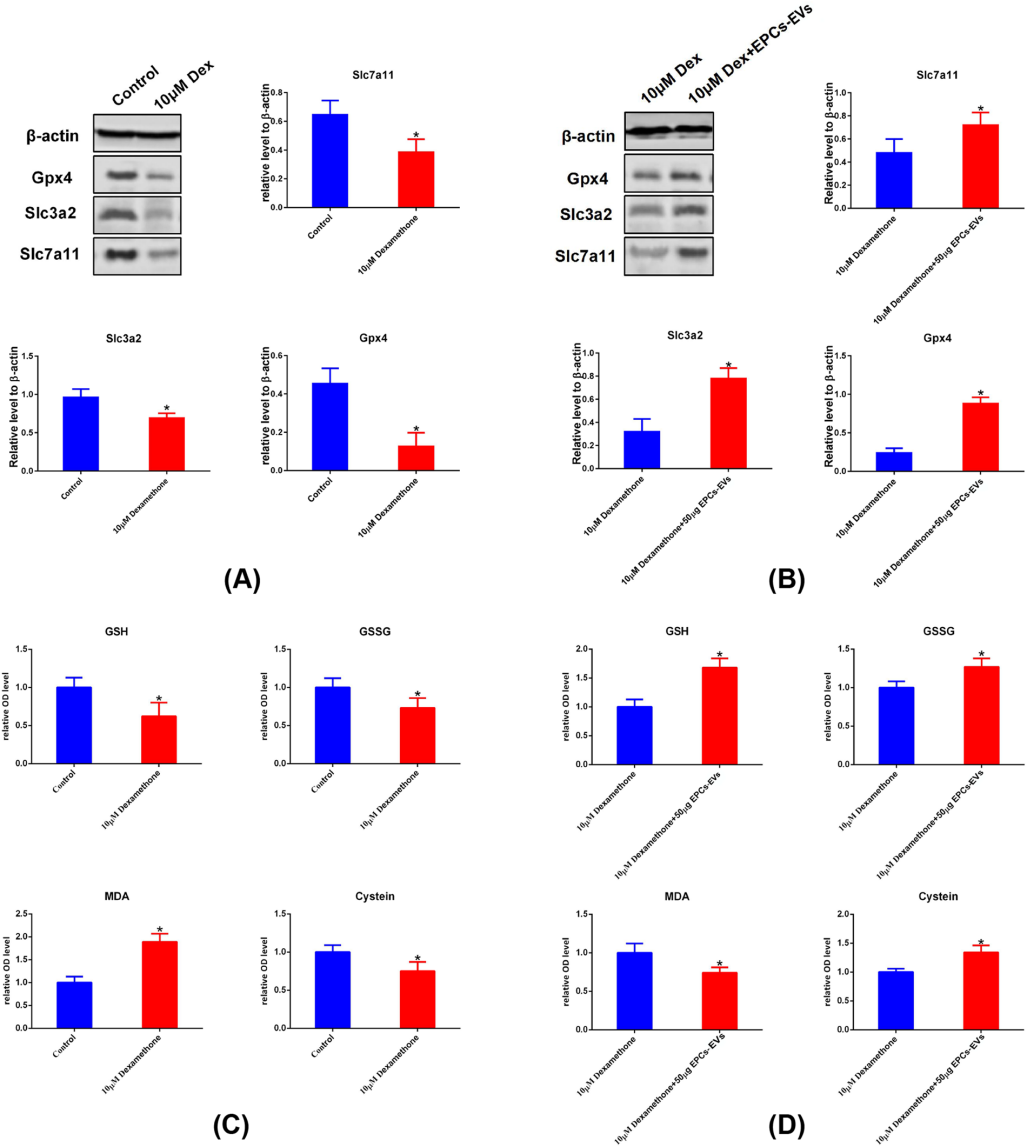
Further confirmation of ferroptotic pathway members and oxidative level detection. Mouse osteoblasts were pre-treated with 10 µM dexamethasone, an equal volume of PBS and a combination of 10 µM dexamethasone and 100 µg EPC-EVs for 72 hours. Cells were lysed and subjected to western blot analysis. (**A**) Representative images and graphs show the expression levels of ferroptotic pathway members, such as GPX4, SLC3A2 and SLC7A11, between the control and 10 µM dexamethasone groups (images were cropped from Fig. S1). (**B**) Represent images and graphs show the expression levels of ferroptotic pathway members, such as GPX4, SLC3A2 and SLC7A11, between the 10 µM dexamethasone + 100 µg EPC-EVs and 10 µM dexamethasone groups (images were cropped from Fig. S1). (**C**) Representative histograms showing the levels of cysteine, GSH, GSSG and MDA between the control and 10 µM dexamethasone groups. (**d**) Representative histograms showing the levels of cysteine, GSH, GSSG and MDA the between 10 µM dexamethasone + 100 µg EPC-EVs and 10 µM dexamethasone groups. *P < 0.05 was considered statistically significant.

To further confirm the differentially expressed ferroptotic mediators that were observed in gene sequencing and gene enrichment analysis, a western blot assay was used to examine the expression level of specific members, such SLC3A2, SLC7A11, and GPX4. In addition, changes in ferroptotic gene expression leads to bioactivity changes in certain enzymes, and relative metabolites are also regulated. Therefore, cysteine, glutathione (GSH), glutathione disulphide (GSSG) and malondialdehyde (MDA), which reflect the state of the ferroptotic pathway, were examined via spectrophotography. These results showed that compared with the control group, the 10 µM dexamethasone group had lower SLC3A2, SLC7A11 and GPX4 expression levels (Fig. 8A). In addition, cysteine, GSH, and GSSG were decreased and MDA was elevated in mouse osteoblasts after 10 µM dexamethasone induction (Fig. 8C). On the other hand, compared with mouse osteoblasts that underwent 10 µM dexamethasone induction, osteoblasts that received a combination of 10 µM dexamethasone and 100 µg (approximately 6.9 × 10^10^ EPC-EVs) of EPC-EV treatment had elevated SLC3A2, SLC7A11 and GPX4; increased cysteine, GSH, GSSG; and decreased MDA (Fig. 8B,D). These results further prove that dexamethasone induced ferroptotic changes and that EPC-EVs could prevent ferroptotic processes by regulating the key mediators and metabolites in the ferroptotic pathway.

### Summary of the functional role of EPC-EVs in the alleviation of SIOP

**Figure 9:**
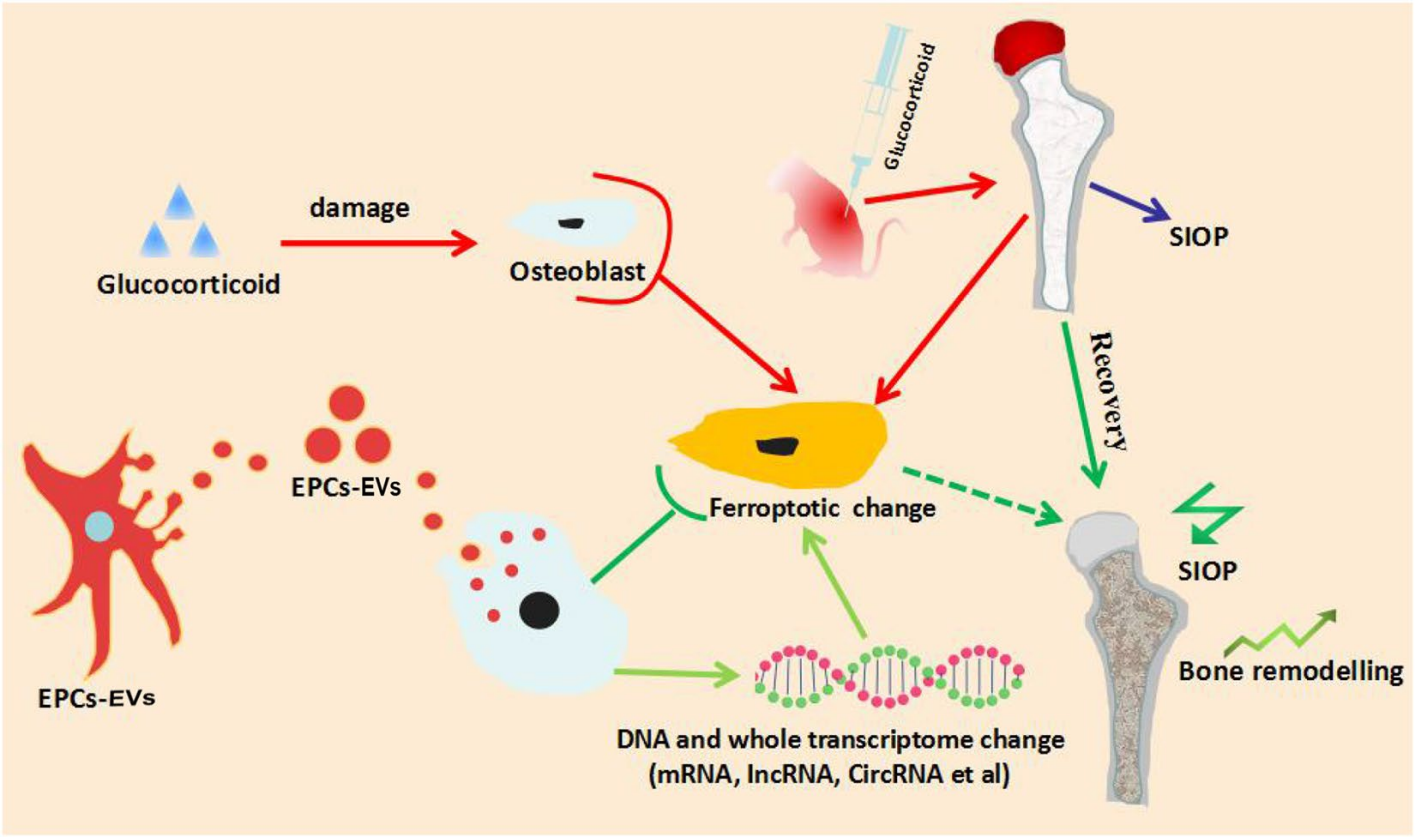
Summary of the functional role of EPC-EVs in the alleviation of SIOP. In the present study, high-dose dexamethasone activated the ferroptotic signalling pathway in mouse osteoblasts. The administration of EPC-EVs triggers alterations in DNA and the transcriptome, inhibiting the activation of the ferroptotic pathway following 10 µM dexamethasone treatment in osteoblasts. The intramuscular delivery of high-dose dexamethasone triggered SIOP in mice, and EPC-EVs alleviated the pathogenesis of SIOP, with improved bone structure parameters.

Based on the present study, BM-EPCs and EPC-EVs were successfully isolated and identified. EPC-EV administration via the portal vein alleviated SIOP in a mouse model with elevated TV, BV, BS, Tb.Th, and Conn-D and reduced the SMI, Tb.Sp and typical histopathological change in osteoporosis. On the one hand, dexamethasone induction activated the ferroptotic pathway based on KEGG enrichment analysis. On the other hand, EPC-EV treatment following dexamethasone induction counteracted the activation of the ferroptotic changes that occurred in osteoblasts from mice that underwent dexamethasone induction. In summary, EPC-EVs were found to have the potential to alleviate mice SIOP by suppressing the ferroptotic pathway in osteoblasts (Fig. 9).

## Discussion

In this study, mouse primary bone marrow-derived EPCs were isolated by density gradient centrifugation and were analysed by using FITC-UEA-I/Dil-Ac-LDL double staining and the immunoblotting of surface markers, such as CD133, CD34, FLK-1 and vWF. Compared with ultracentrifugation, decreased time, lower pre-experiment amounts of sample and higher extraction rates of extracellular vesicles were required by an exosomal isolation kit, which provided us with a powerful tool to examine the real-time functional properties of EPCs^29–32^. The characterization results showed that the morphology of the isolated samples was similar to the characteristics of extracellular vesicles, and the surface markers, such CD9, CD63, and CD81, were highly expressed in the isolated samples compared with in EPCs with the same loading amounts, which met the requirements for the subsequent tests.

Micro-CT analysis showed that EPC-EVs improved SIOP-related bone parameters, such TV, BV, BS, Tb.Th, Tb.Sp, Conn-D and the SMI. In addition, the cross-sectional images of the coronal and sagittal planes showed that EPC-EVs not only alleviated osteonecrosis but also increased the amount of trabecular bone and the bone density to some extent. Histopathological examinations showed that EPC-EVs reduced the formation of necrotic tissue in the femoral head and increased the amount of cancellous bone, trabecular bone, the density of collagen fibres and the abundance of stromal cells. Based on the key pathological characteristics of SIOP, potential treatments that aim to improve the blood supply close to the lesion and to promote the regeneration of bone tissue may contribute to the alleviation of this syndrome as well as to the partial recovery of the structure of damaged bone tissue. Since completely destroyed blood vessels cannot be repaired, the promotion of angiogenesis to compensate for the devastated vessels is highly needed^33^. Once there is a sufficient blood supply, multiple cells would have enough nutrients to support their differentiation and proliferation. On the other hand, osteogenesis also contributes to the restoration of SIOP, which requires the activation of osteoblasts and osteoclasts and the coordination of the activation and differentiation of bone marrow MSCs^34^.

Several studies have reported that EPC-EVs were able to promote skin wound healing, repair pulmonary vascular injuries and repair myocardial damage by promoting angiogenesis, facilitating the proliferation of fibroblasts in the skin, heart and lung as well as increasing the migration and differentiation capacity of MSCs^35–37^. The treatment of osteoporosis requires the enhancement of the bone remodelling process, which involves the attachment of osteoclasts to the obsolete bone, the release of acidic products and proteases to dissolve minerals and to digest the bone matrix, and the formation of a bone resorption lacuna^38^. Subsequently, osteoblasts migrate to the site of absorption and secrete bone matrix. Finally, the bone matrix mineralizes to form new bone^39^. Osteoblasts play a vital role in bone regeneration and lead the synthesis, secretion and mineralization of the bone matrix^40^. Therefore, we tried to explore the effect of EPC-EVs on osteoblasts by investigating the transcriptomic differences and the subsequent bio-productivity of osteoblasts. In this study, we found that mouse osteoblasts were able to internalize fluorescence-labelled EPC-EVs in a dose-dependent manner. On this basis, we used whole transcriptome sequencing to explore the internal alterations in gene expression following dexamethasone or EPC-EV administration after dexamethasone pre-treatment.

Ferroptosis is a recently discovered type of programmed cell death. Unlike apoptosis, autophagy, and necrosis^41^, ferroptosis is caused by the inhibition of GPX4 and X^c−^, which leads to the inhibition of cystine metabolism, elevated lipid peroxidation and iron metabolism dysfunction, producing large amounts of ROS and triggering cell death^42^. Glucocorticoids induced oxidative stress in various cell types, such as osteoblasts and hippocampal cells, which was accompanied by a significant elevation in the ROS level^43^. This oxidation and antioxidation imbalance, together with the abnormal activation of the MAPK, Wnt and P38 signalling pathways, led to apoptosis and autophagy in various cells, such as osteoblasts, osteoclasts and nerve cells^44^. The difference in the doses of glucocorticoids may affect the expression of GPX1, GPX4, MnSOD, SOD1, SOD2 and other antioxidants in pregnant animals at different time points^45^. In addition, high-dose glucocorticoids can also reduce the expression of SOD and GPX in cells such as macrophages and skeletal muscle^46^. Clinical trials have confirmed that patients with long-term hormone therapy, postmenopausal therapy, and primary osteoporosis have low levels of GPX and SOD^47^. To the best of our knowledge, there have been no studies on ferroptosis and steroid-induced orthopaedic disorders, and no studies have confirmed that glucocorticoids can induce the activation of the ferroptotic pathway in osteoblasts.

Based on the present study, dexamethasone treatment activates the ferroptotic signalling pathway, resulting in the downregulation of GPX4 and system X^c−^ in osteoblasts. On the other hand, the observed elevation of MDA and ROS, and decreased cysteine production suggesting that dexamethasone increased lipid peroxidation levels, causing metabolic disorders involving glutamate and cysteine, which indicates that this hormone can induce ferroptotic changes in osteoblasts. EPC-EVs were able to upregulate GPX4, systemX^c−^ and cysteine levels while reducing MDA and ROS production. Taken together, these results indicated that EPC-EVs may exert their function by antagonizing the glucocorticoid-induced activation of the ferroptotic pathway.

Steroid-induced osteoblast apoptosis and autophagy have been well documented in many studies, and there is sufficient evidence for these relationships^47,48^. In the present study, the characteristic changes in ferroptosis in mouse osteoblasts were not fully examined. Our bioinformatics analysis showed that apoptosis and autophagy-related pathways were also affected by dexamethasone treatment, which was consistent with a literature report^49^. Therefore, we believe that the ferroptosis, apoptosis and autophagy signalling pathways, which are related to cell damage and death, played an essential role in glucocorticoid-mediated osteoblast damage. However, glucocorticoid-induced cell damage cannot be explained by apoptosis and autophagy alone. Similar to the observed therapeutic effect of EPC-EVs on osteoblasts, the bioinformatics analysis showed that there were other signalling pathways, such as osteoblast differentiation and the hedgehog pathway, that were involved. Further investigations focusing on other related pathways are needed to obtain a more detailed understanding of the underlying molecular mechanisms.

Extracellular vesicles are tiny vesicles that contain large amounts of functional proteins, mRNA, long non-coding RNAs (lncRNAs) and miRNA (microRNA), which are indispensable for the transcriptional regulation of osteoblasts by EPC-EVs after their uptake and internalization^50^. miRNA, a group of small non-coding RNA that are approximately 22 nucleotides long, functions in RNA silencing and post-transcriptional regulation via base-pairing with complementary sequences and targeting mRNA molecules. miRNA-17-92, miRNA-9 and miRNA-137 have been reported to have anti-ferroptotic effects via the A20-ACSL4 axis in epithelial cells and via GOT1 and SLC1A5 in melanoma^51–53^. In addition, lncRNA, a type of RNA over 200 nucleotide long that are not translated into protein, was previously found to have rare biological functions, while lncRNA were widely reported to be involved in transcriptional regulation in the present study. LncRNA P53RRA was found to promote ferroptosis in lung cancer via the nuclear sequestration of p53, and LINC00336 was shown to inhibit ferroptosis by functioning as a competing endogenous RNA^54,55^. Therefore, the main functional substance in specific types of extracellular vesicles and their regulatory mechanisms are still ambiguous and have attracted attention worldwide. A detailed understanding of the biological mechanism will help us to find new targets for the treatment of SIOP. In addition, the influence and regulation of EPC-EVs on the activation of pathways involved with other types of programmed cell death in osteoblasts still need to be elucidated. At the same time, the pathogenesis of SIOP involves many kinds of cells, such as MSCs and osteoclasts. The multi-omics bioinformatics analysis of EPC-EVs in diverse participants and the exchange and transmission of information between cells need to be further explored.

## Materials and Methods

### Isolation and identification of BM-EPCs

A total of 20 male Kunming (KM) mice (70 ± 20 g) were obtained from the Laboratory Animal Center of Anhui Medical University (Hefei, China). The present study was approved by the Medical Ethics Committee of the Academic Committee at Anhui Medical University. Mice were sacrificed by carbon dioxide asphyxiation, and the surface of the skin was disinfected with 70% alcohol. The lower limbs of the mice were removed with scissors, and the muscles and soft tissues attached to the bones were removed, leaving clean, bilateral femurs. Scissors were used to remove both ends of the long bone, and the medullary cavity was rinsed with a syringe. The rinsing solution was transferred to a 50-ml tube, and bone marrow cells were collected by centrifugation at 2000 g for 5 minutes. The collected bone marrow cells were then resuspended in 6 ml of PBS in a 15-ml tube, and 8 ml of warm Histopaque-1083 (Sigma, Missouri, USA) was added and centrifuged at 11,000 g for 20 minutes. The liquid was divided into four layers after centrifugation, the uppermost layer of PBS was discarded, and the pink cell layer underneath was transferred to a new 15-ml Eppendorf (EP) tube, resuspended in 5 ml PBS, and centrifuged at 2000 g for 5 minutes. After centrifugation, the supernatant was removed, and the cells were resuspended in 5 ml endothelial cell growth basal medium-2 (EBM-2) (Lonza, Minnesota, USA) with 3% foetal bovine serum (FBS). Dil-Ac-LDL and FITC-UEA-I (Thermo Fisher, Massachusetts, USA) were utilized to identify bone marrow-derived EPCs via double staining. Mature EPCs were positive for both markers. The isolated cells were expanded until they reached 75% confluence and 10 µM Dil-Ac-LDL was added to the medium and was incubated for 4 hours. Then, the sample was fixed with 10% paraformaldehyde for 10 minutes and was incubated with 10 µM FITC-UEA-I for 1 hour at room temperature. Then, the cells were observed with confocal laser scanning microscopy (FITC: excitation wavelength, 488 nm; emission wavelength, 522 nm; Dil: excitation wavelength, 552 nm; emission wavelength, 569 nm). CD133, CD34, vWF, and FLK-1 are typical surface markers of EPCs, and flow cytometry was utilized to detect these biomarkers. Briefly, BM-EPCs were cultured to the logarithmic growth phase and were transferred to 12-well plates with 5*10^5^ cells per well. One FITC-labelled fluorescent primary antibody was added to each well (1:3000) and was incubated for 60 minutes. Next, the medium was discarded, and the cells were washed 3 times with PBS. The cells were digested and resuspended t in PBS waiting for flow cytometry analysis (FITC: excitation wavelength, 488 nm; emission wavelength, 522 nm). Cells that were not incubated with an antibody were used as a control for fluorescence compensation.

### Isolation and characterization of EPC-EVs

The isolation and characterization of extracellular vesicles were conducted according to the guidance of Minimal information for studies of extracellular vesicles 2018 (MISEV2018)^56^. *Extracellular vesicle isolation* EPC-EVs were isolated with Umibio exosome isolation kits (Umibio, cat. no: UR52121, Shanghai, China) according to the manufacturer’s guidelines. Briefly, EPCs were cultured in medium with extracellular vesicle-free FBS. Fifty millilitres of culture media was harvested and centrifuged at 3000 × g and 4 °C for 10 minutes and at 10,000 × g and 4 °C to remove cells and debris. The supernatant was transferred to a new container without disturbing the pellet. Then, 25 ml of total extracellular vesicle isolation reagent was added, and the reaction mixture was mixed by rotation. The mixture was incubated at 4 °C for two hours and was then centrifuged at 10,000 g for 1 hour at 4 °C. The supernatant was discarded, and the pellet was resuspended with 1000 µl of 1x PBS. The isolated extracellular vesicles were stored at −80 °C for long-term storage.

#### Immunoblotting

Immunoblotting was utilized to detect the surface markers of extracellular vesicles, such as CD81, CD63 and CD9. Briefly, 100 µl of isolated extracellular vesicles were lysed with radioimmunoprecipitation assay (RIPA) lysis buffer and then centrifuged at 12,000 g for 30 minutes to extract the supernatant. The quantitation of isolated extracellular vesicles was conducted with a BCAkit (Beyotime, Shanghai, China). The proteins were denatured via heating at 100°C for 5 minutes with 1/5 of the sample volume of loading buffer (Beyotime, Shanghai, China). Then, 20 µg was loaded onto 10% SDS-PAGE gels and was transferred to PVDF membranes. The membrane was blocked with fat-free milk and was incubated with the appropriate primary antibodies, such as CD81, CD63 and CD9, overnight at 4 °C. Then, the samples were washed again and incubated with an appropriate secondary antibody for 1 hour at room temperature. Membranes were washed with PBS and were detected with a gel imager with a luminol substrate solution.

#### NTA

NTA was utilized to detect the particle size of the extracellular vesicle sample. Briefly, 20 µl of prepared extracellular vesicles were diluted in 1000 µl with deionized water. The diluted sample was slowly and carefully loaded into a 1-ml syringe and into the loading tube of the nanoparticle tracking system (PMX110, Zetaview). Analysis was started once the pictures of the particle on the screen become stable (Fig. S4).

#### Bio-transmission microscopy

Histological features of extracellular vesicle samples were observed via a transmission electron microscope (Tecnai G2 Spirit Biotwin, FEI, Hong Kong). Briefly, 20 µl of the extracellular vesicle sample was added to a copper-mesh grid, and the liquid was left on the copper-mesh grid for 4 minutes. The sample was negatively stained with 2% uranyl acetate solution for 4 minutes and was then dried off with filter paper. The copper mesh was placed under a 120-kV bio-transmission electron microscope and was photographed.

### Animal modelling and grouping

The present study was approved by the Medical Ethics Committee of the Academic Committee at Anhui Medical University, and all methods were carried out in accordance with the relevant guidelines and regulations of animal ethical standards. Thirty KM mice (male, 70 ± 20 g) were obtained from the Laboratory Animal Center of Anhui Medical University (Hefei, China) and were randomly divided into 3 groups, which included the normal group, the SIOP group, and the extracellular vesicle treatment group. Mice in both the SIOP model group and the extracellular vesicle treatment group were intramuscularly injected with 20 mg/kg dexamethasone into the hind leg five times a week for eight weeks in total. Eight weeks later, the extracellular vesicle treatment group was given 50 μg of extracellular vesicles (approximately 3.45 × 10^10^ vesicles) via the tail vein. Both the control group and the model group were injected with the same volume of normal saline once a week for four weeks and were sacrificed after another two weeks. Finally, 1 ml of blood was taken by retro-orbital bleed, and the serum was stored at −20 °C. The mice were sacrificed by cervical dislocation. The muscles and soft tissues were removed with scissors and forceps, and the whole femur was recovered. The samples were fixed in a 4% paraformaldehyde solution for 24 hours and then were stored in a 70% alcohol solution for subsequent experiments.

### Micro-CT and histopathological analysis

After the completion of the relevant experiments, the mice were sacrificed by cervical dislocation. The skin and muscles of the bilateral lower limbs were carefully removed with scissors and forceps until the femur was fully exposed. The soft tissue was cut at both ends of the femur, and the femurs were fully separated on both sides without damaging the femoral head. The bilateral femurs of each mouse were then placed in a 25-ml cuvette and were fixed in 4% paraformaldehyde for 24 hours. The femurs of each group were equally divided into two groups. One group was fixed with 4% paraformaldehyde, repeatedly washed and placed in 70% alcohol for storage. The whole femur was directly subjected to micro-CT (SkyScan 1176, Bruker, Beijing) scanning after drying off the femur with filter paper. Following reconstruction, the region of interest (ROI) was set as the whole femur. The osteoporosis-based measurements for the ROI of each femur were manually determined with CTan 1.10 analysis software with parameters including TV, BS, BV, BS/TV, BV/TV, BS/BV, Conn-DTb.sp, Tb.Th, and the SMI. The severity of osteoporosis was positively correlated with BS, BV, BS/TV, BV/TV, Tb.Th and Conn-D and was negatively correlated with Tb.sp and the SMI. Sectional coronal and sagittal images and cross-sections were collected and analysed. According to the scanning results from micro-CT, the 2-D images were reconstructed into 3-D images.

The other group of femurs was decalcified using EDTA for 3–5 days after fixation. In the decalcification process, the flexibility was evaluated daily, and the hardness was evaluated by acupuncture until the specimen was very flexible and the sample could be easily penetrated by the needle. The decalcified specimens were then subjected to paraffin embedding and sectioning, followed by HE staining and Masson staining. Briefly, the sections were dehydrated with ethanol, dewaxed with xylene, and stained for 10 minutes after haematoxylin staining, followed by eosin staining for 20 s. After rinsing, the xylene was transparent after 5 minutes, and the slide was cover slipped. For Masson staining, the sections were dewaxed and stained with haematoxylin for 5 minutes, washed three times with deionized water and stained with Masson dye for 5–10 minutes. Then, the samples were immersed in a 2% glacial acetic acid solution, and was hydrated with 10% molybdic acid for 3–5 minutes. After aniline blue staining for 5 minutes and gradient alcohol washes, the slide was sealed with a neutral gum and was observed under an optical microscope.

### Cell culture and EV internalization

Mouse osteoblasts (MC3T3-E1 Subclone 14) were purchased from an American Type Culture Collection (ATCC) cell bank (ATCC CRL-2594) and were cultured in 1640 medium at 37 °C in a 5% CO_2_ atmosphere. Extracellular vesicles were labelled with a PKH26 fluorescent labelling kit (Sigma, USA) according to the protocol. Briefly, 100 μg of extracellular vesicles (approximately 6.9 × 10^10^ EVs) and 4 μl of PKH26 dye were mixed with 1 ml Diluent C, were incubated for 5 minutes, and the reaction was stopped with 1% BSA. Excess dye was removed by centrifugation at 200,000 g for 30 minutes with a 30% sucrose/D_2_O density gradient. The sucrose was then removed using a 100-kD ultrafiltration tube to obtain PKH26-labelled extracellular vesicles. PKH26-labelled extracellular vesicles at 10, 20, and 50 μg/ml (approximately 0.69 × 10^10^, 1.38 × 10^10^ and 3.45 × 10^10^ extracellular vesicles) were co-cultured with cells for 3 hours. Cells were fixed with 4% paraformaldehyde, and nuclei were stained via 5 μM Hoechst 33342. Images were taken with a fluorescence microscope, and images were processed and analysed with ImageJ software.

### RNA-seq and bioinformatics analysis

Mouse osteoblasts were cultured and divided into 3 experimental groups (n = 2) that were treated with 10 µM dexamethasone, 10 µM dexamethasone + 100 µg EPC-EVs or 100 µl PBS for 72 hours (5*10^6^/sample and 6 samples in total). All cells were trypsinized, and total RNA was extracted using TRIzol (Invitrogen, USA). RNA purity and integrity were tested using a NanoDropPhotometer® spectrophotometer (IMPLEN, CA, USA) and an RNA Nano 6000 Assay Kit with a Bioanalyzer 2100 system (Agilent Technologies, CA, USA). Finally, the RNA concentration was assessed using a Qubit® RNA Assay Kit in a Qubit® 2.0 Fluorometer (Life Technologies, CA, USA). The clustering of the index-coded samples was performed on a cBot Cluster Generation System using a TruSeq PE Cluster Kit v3-cBot-HS (Illumina) according to the manufacturer’s specifications. To sequence the libraries on an Illumina HiSeq 4000 instrument, the barcoded libraries were mixed, denatured in NaOH to generate single-stranded DNA, captured on an Illumina flow cell, amplified *in situ*, and sequenced for 150 cycles for both ends on an Illumina HiSeq 4000 instrument.

In-house Perl scripts were used to process the raw data (raw reads) in a FASTQ format. Clean data (clean reads) were obtained by removing reads containing adapters and poly-N sequences, and low-quality reads were also removed from the raw data.

The first step of analysing the processed RNA-seq data was to align the reads to a reference genome. The index of the reference genome and the gene model annotation files (obtained from GEO website) were used for annotation. HISAT2 v2.1.0 was used to align the clean reads to the reference genome. HISAT2 was run with ‘–rna-strandness RF’, and the other parameters were set to the default settings. HTSeq v0.6.1 was used to count the read numbers mapped to each gene. Gene fragments Per Kilobase of transcript per Million mapped reads (FPKMs) were calculated by summing the FPKMs of the transcripts in each gene group. Finally, the differential gene expression analysis of two conditions was performed using the DESseq R package (1.26.0). Genes with a log2-fold change of more than 1.5 or less than −1.5 and with a P-value < 0.05 were considered DEGs.

The calculation of mRNA FPKMs in each sample was performed using StringTie (v1.3.3) by summing the FPKMs. Differential mRNA expression analysis of two conditions/groups was performed using the DESeq R package (1.26.0). A log2-fold change of more than 1 or less than −1 and a P-value < 0.05 were considered differentially expressed mRNAs.

GO term enrichment is a widely used bioinformatics tool for interpreting genes sets with a set of predefined terms to better understand the underlying biological processes of some genes. GO enrichment analysis was performed with the Database for Annotation, Visualization, and Integrated Discovery (DAVID) database to predict more functions and mechanisms of DEGs and mRNA. Significant GO term were defined as those with a P-value < 0.05. In addition, based on the KEGG database, we used KEGG Orthology Based Annotation System (KOBAS) v3.0 software to perform pathway analysis for DEGs and mRNA^57–59^. We chose a cut-off based on the P-value, and all pathways with a P-value less than 0.05 were included in the subsequent analysis. Use of KEGG imaginary was approved with the official permission from Kanehisa laboratory.

### Western blotting

Western blotting and the analysis of the blot results was conducted as previously reported^27^. In brief, cells were lysed and centrifuged at 12,000 g for 10 minutes at 4 °C. The supernatants were denatured at 100 °C for 5 minutes and mixed with a 1/5 volume (corresponding to the volume of the supernatants) of loading buffer (Beyotime, China). Then, 30-µg protein samples were loaded into each well in a 10% SDS-polyacrylamide gel and were subjected to electrophoresis. Once the proteins of diverse molecular weights were sufficiently separated, the proteins were transferred to PVDF membranes (Millipore, USA). Next, the PVDF membranes with the transferred proteins were blocked with 5% nonfat milk diluted in TBST for 2 hours at 25 °C and were then incubated with primary antibodies, such as SLC7A11, SLC3A2, GPX4, GSS, GCLC, ACSL4 (Cell Signaling Technology, USA) and β-actin (Beyotime, Shanghai, China), with dilutions of 1:1500, 1:2000, 1:2000, 1:2500, 1:3000 and 1:10,000, respectively, at 4 °C for 12 hours. Next, the membrane was washed again with TBST and was incubated with a 1:10,000 dilution of horseradish peroxidase (HRP)-labelled anti-rabbit IgG (Beyotime, Shanghai, China) for 1 hour at 25 °C. Finally, the respective protein bands were visualized using enhanced chemiluminescence reagents (BOSTER, Wuhan, China).

### ELISA

Before sacrificing the animals, 1 ml of blood was collected from mice in different groups by retro-orbital bleeds. Whole blood from each mouse was transferred into a 2-ml tube and was centrifuged at 2000 g for 20 minutes to obtain as much clear serum as possible. The supernatant was removed, transferred to another 2-ml tube and stored at −20 °C for future usage. Cysteine was detected with a cysteine detection kit (Bost Biotechnology, China) according to the corresponding protocol. Briefly, 0.1 ml of liquid was added to a 1-ml EP tube, and 0.9 ml of phosphotungstic acid reagent was added to the same tube. The mixture was centrifuged at 8000 g for 4 minutes at 4 °C to obtain the supernatant. Then, the supernatant was loaded into a new tube and was analysed with a microplate reader (BIO-TEK ELX-800, USA) at a wavelength of 600 nm. Thiobarbituric acid-reactive substances (TBARS) assays were performed to assess lipid peroxidation. MDA, a product of lipid peroxide degradation, combined with thiobarbituric acid (TBA) to form a pink product. According to the manufacturer’s instructions, an MDA detection kit (Vazyme, China) was used to determine the MDA content. Briefly, the serum of mice was blended with various reagents and was incubated in 95 °C water for 40 minutes and was subsequently centrifuged at 3000 rpm for 10 minutes. Next, the supernatant was collected, and its absorbance was measured on a microplate reader at 532 nm. 5,5′-Dithiobis-2-nitrobenoic acid (DTNB) reacts with GSH to form a complex with a characteristic absorption peak at 412 nm, and its absorbance is proportional to the GSH content. 2-Vinylpyridine inhibits the original GSH in the sample and then uses glutathione reductase to reduce GSSG to GSH, thereby determining the content of GSSG. The glutathione peroxidase and glutathione reductase ratio was determined by a Micro Oxidized GSSG Assay Kit and Micro Reduced GSH Assay Kit (Solabio, China) according to the provided protocols.

### Statistical analysis

Apart from bioinformatics analysis, the data in the present study were analysed with GraphPad Prism 4.0. The data are presented as the mean ± the standard deviation (SD) of triplicate experiments. A t test was used to compare two groups. P < 0.05 was considered statistically significant.

## Supplementary information

Dataset 1
