# Supplementary material for: Extracellular vesicles from endothelial progenitor cells prevent steroid-induced osteoporosis by suppressing the ferroptotic pathway in mouse osteoblasts based on bioinformatics evidence
Source: Sci Rep. 2019 Nov 6;9:16130. doi: 10.1038/s41598-019-52513-x (PMC6834614; doi:10.1038/s41598-019-52513-x)
Supplement: Supplementary file 1 — Dataset 1 [file 41598_2019_52513_MOESM1_ESM.pdf]

Supplementary Information

Extracellular vesicles of endothelial progenitor cells prevent steroid-induced osteoporosis via suppression of ferroptotic pathway in mice osteoblast based on bioinformatic evidence.

Jinsen Lu[1,*] , Jiazhao Yang[1] ,Yongshun Zheng[1], Xiaoyu Chen[2], Shiyuan Fang[1,*]

[1]Department of Orthopaedics, Anhui Provincial Hospital, Anhui Medical University, Lujiang Road No.17, 230001 Hefei, China.

[2]Department of histology and embryology, Anhui Medical University, Meishan Road No. 81, 230032 Hefei, China.

*Corresponding at: Prof. Shiyuan Fang, E-mail: fangshiyuan@126.com;

Dr Jinsen Lu, E-mail: lujinsen@stu.ahmu.edu.cn

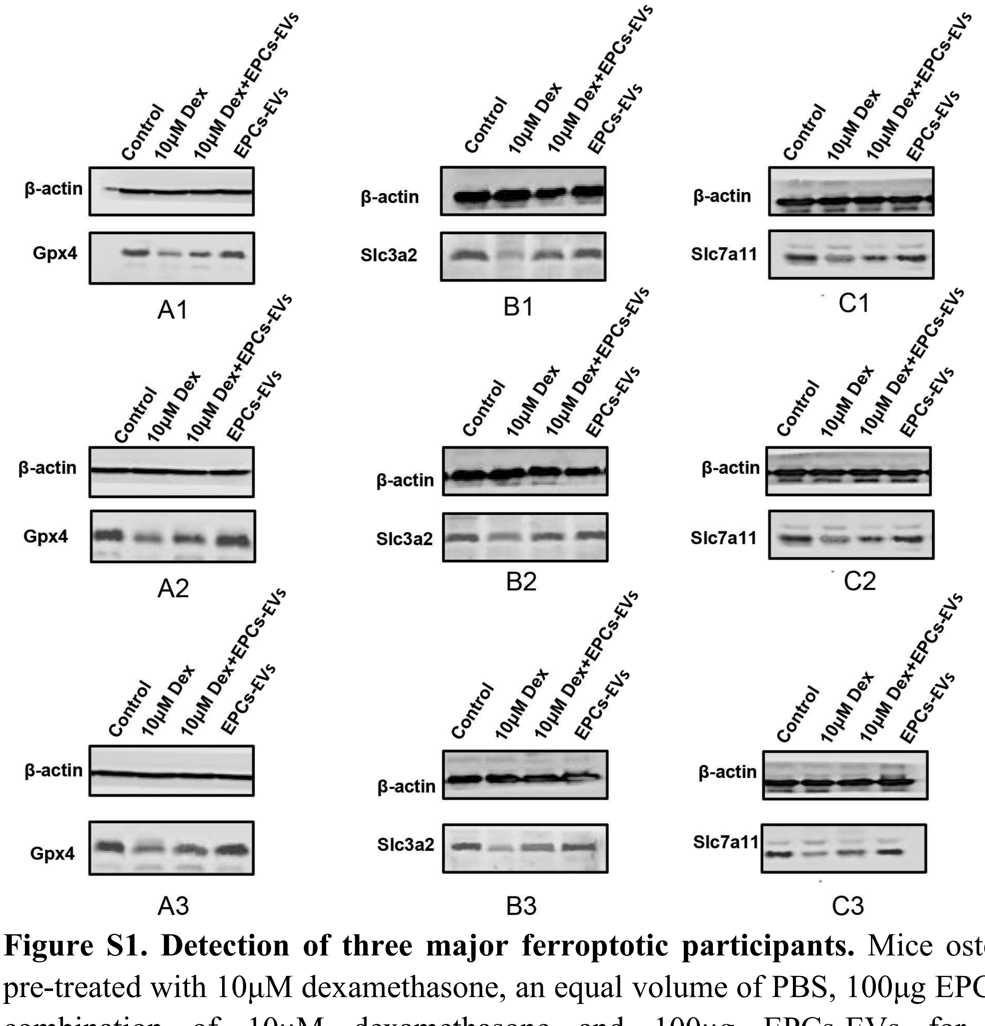

Figure S1. Detection of three major ferroptotic participants. Mice osteoblast was pre-treated with 10μM dexamethasone, an equal volume of PBS, 100μg EPCs-EVs and combination of 10μM dexamethasone and 100μg EPCs-EVs for 72 hours. Cells were lysed and receiving western blot analysis. Represent images and graphs show the expressive level of ferroptotic participants including (A1-3) GPX4, (B1-3)SLC3A2 and (C1-3) SLC7A11 between control, 10uM Dexmethone group, 10μM dexamethasone+100μg EPCs-EVs and 100μg EPCs-EVs group in three repeated experiments.

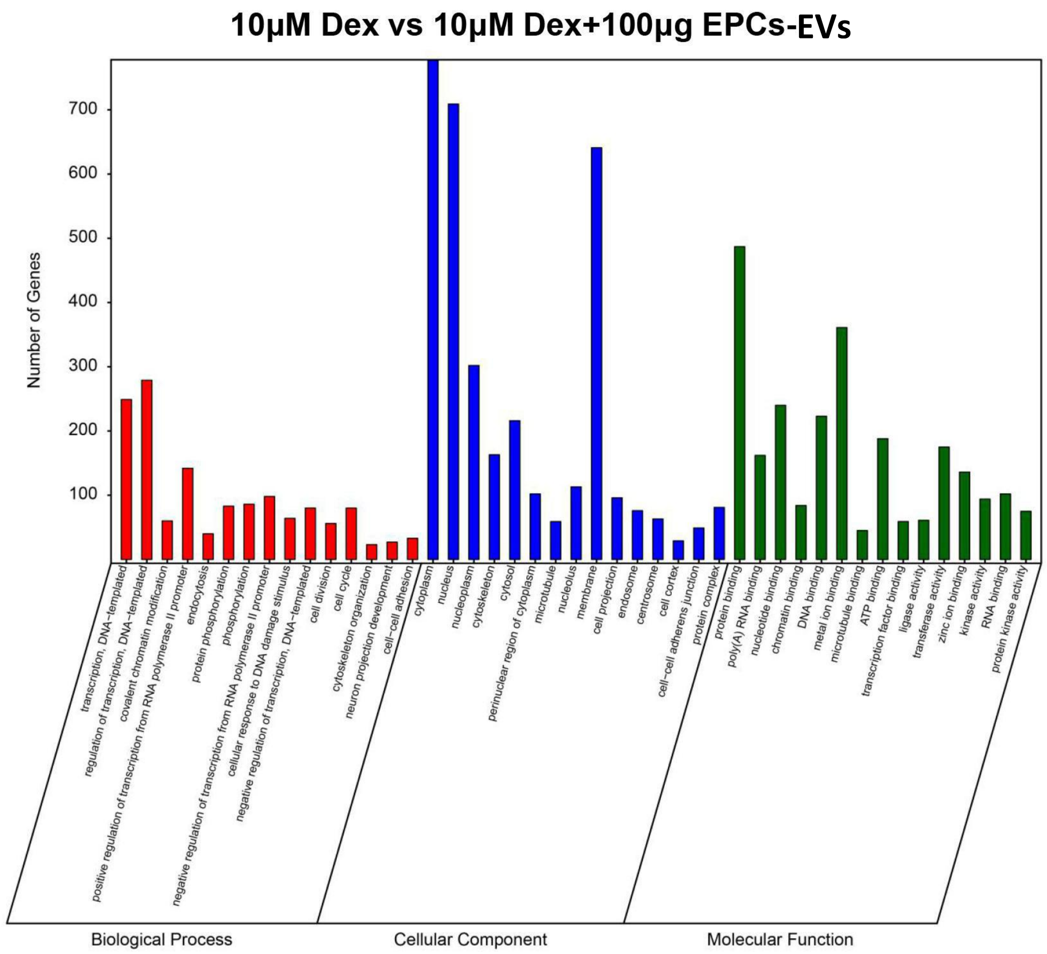

Figure S2. GO enrichment analysis of differentially expressed gene.

Osteoblast was pretreated with 10μM dexamethone and combination of 10μM dexamethone and 100μg EPCs-EVs for 72 hours. Total RNA was extracted and detected via RNA-sequencing. The number of the differentially expressed gene was mapped into specific pathways in GO database in terms of 3 aspects including molecular function, cellular component, and biological process.Y-axis was determined via the number of genes in certain pathway. Top 10 pathway were listed in X-axis.

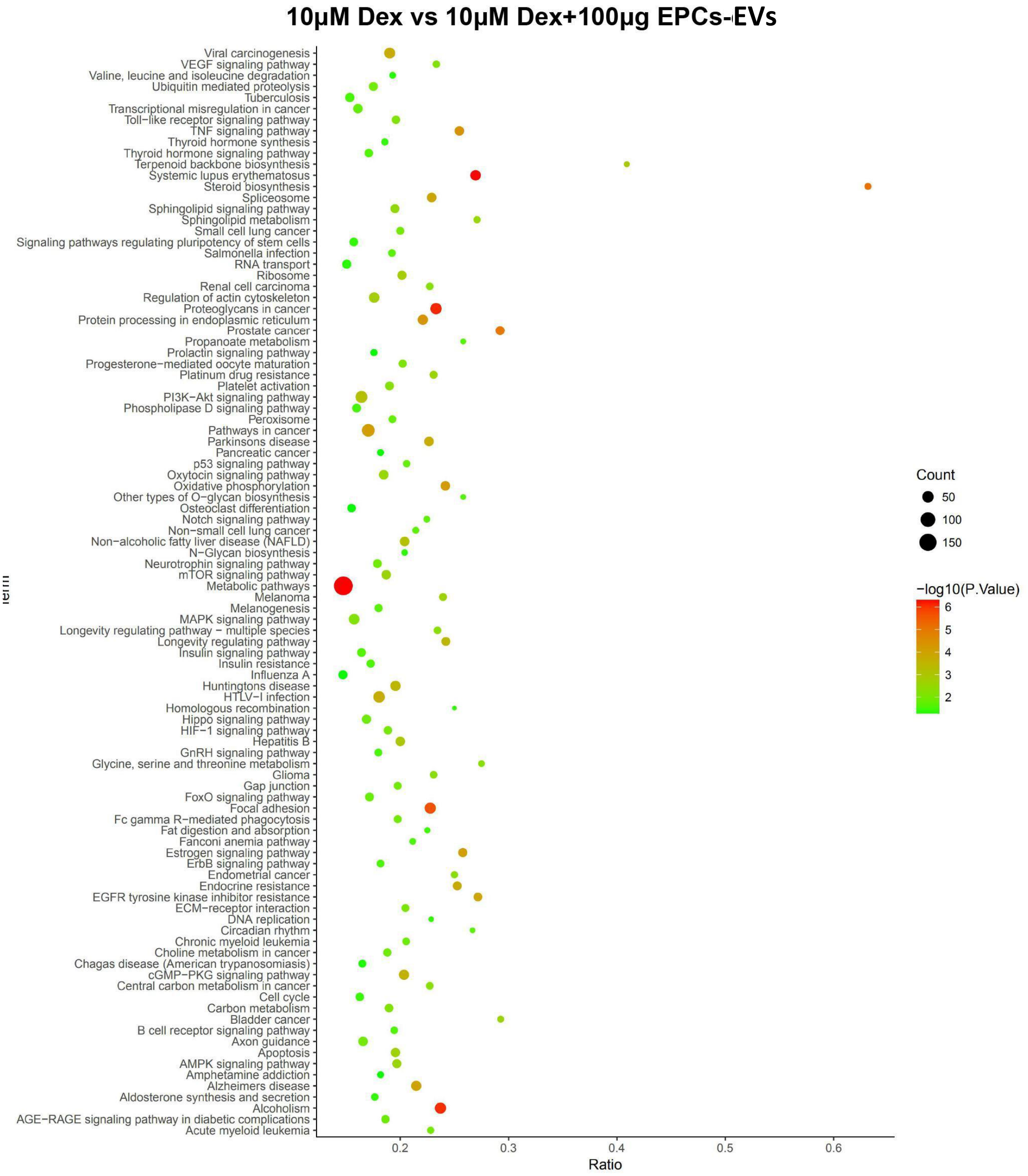

Figure S3. KEGG enrichment analysis of differentially expressed gene. Osteoblast was pretreated with dexamethasone and a combination of dexamethasone and EPCs-EVs for 72 hours. Total RNA was extracted and detected via RNA-sequencing. The number of the differential expressed gene was mapped into specific pathways in KEGG database. Y-axis shows the top 55 pathway involved in KEGG database. The number of genes mapped to a certain pathway was expressed as the size of dot. The intensity of difference was expressed as the color from green to red (PValue).

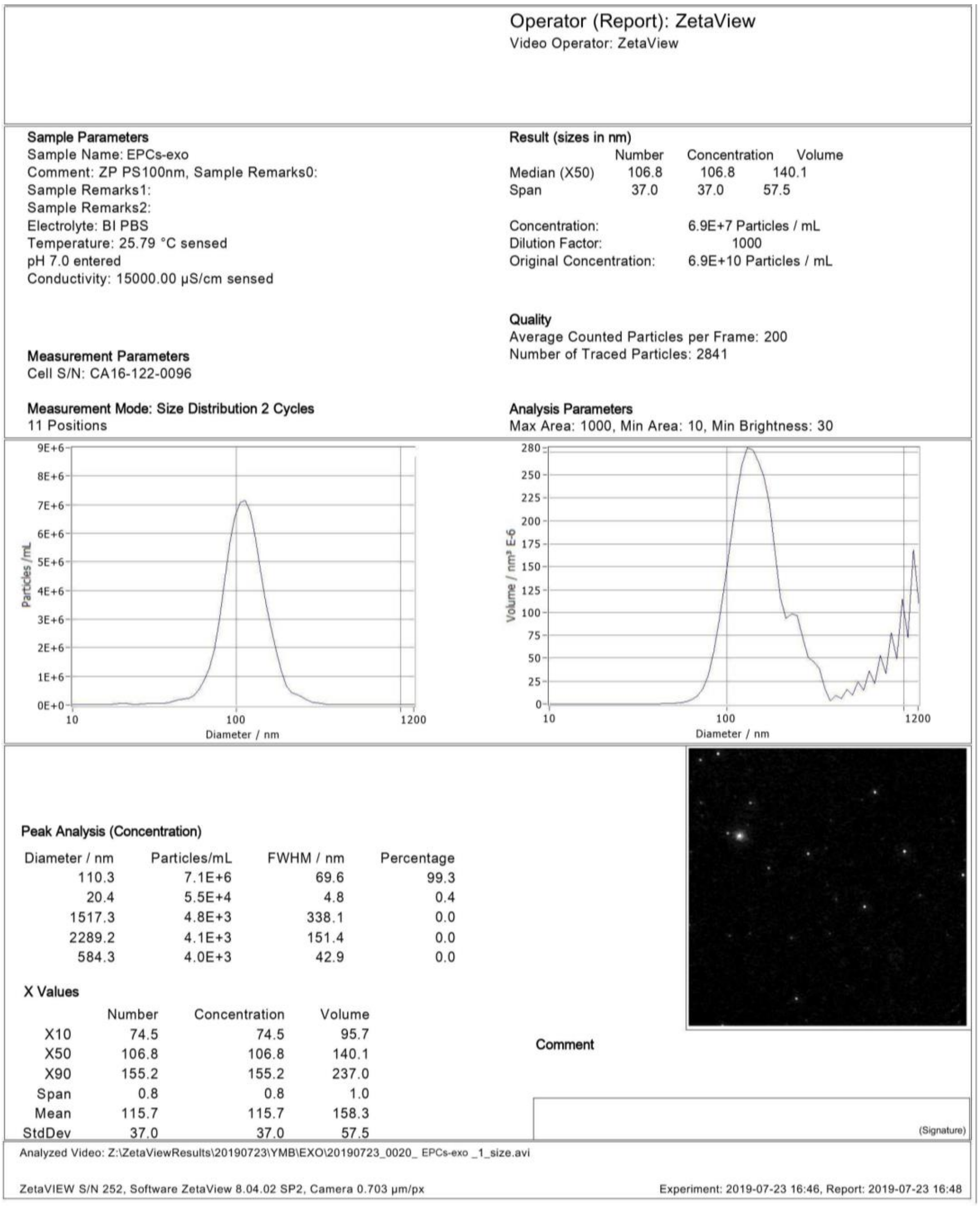

Operator (Report): ZetaView
Video Operator: ZetaView

Sample Parameters
Sample Name: EPCs-exo
Comment: ZP PS100nm, Sample Remarks0:
Sample Remarks1:
Sample Remarks2:
Electrolyte: BI PBS
Temperature: 25.79 °C sensed
pH 7.0 entered
Conductivity: 15000.00 µS/cm sensed

Measurement Parameters
Cell S/N: CA16-122-0096

Measurement Mode: Size Distribution 2 Cycles
11 Positions

Result (sizes in nm)

	Number	Concentration	Volume
Median (X50)	106.8	106.8	140.1
Span	37.0	37.0	57.5

Concentration: 6.9E+7 Particles / mL
Dilution Factor: 1000
Original Concentration: 6.9E+10 Particles / mL

Quality
Average Counted Particles per Frame: 200
Number of Traced Particles: 2841

Analysis Parameters
Max Area: 1000, Min Area: 10, Min Brightness: 30

Peak Analysis (Concentration)

Diameter / nm	Particles/mL	FWHM / nm	Percentage
110.3	7.1E+6	69.6	99.3
20.4	5.5E+4	4.8	0.4
1517.3	4.8E+3	338.1	0.0
2289.2	4.1E+3	151.4	0.0
584.3	4.0E+3	42.9	0.0

X Values

	Number	Concentration	Volume
X10	74.5	74.5	95.7
X50	106.8	106.8	140.1
X90	155.2	155.2	237.0
Span	0.8	0.8	1.0
Mean	115.7	115.7	158.3
StdDev	37.0	37.0	57.5

Comment

(Signature)

Analyzed Video: Z:\ZetaViewResults\20190723\YMB\EXO\20190723_0020_ EPCs-exo _1_size.avi

ZetaVIEW S/N 252, Software ZetaView 8.04.02 SP2, Camera 0.703 µm/px

Experiment: 2019-07-23 16:46, Report: 2019-07-23 16:48

Figure S4. NTA overall report

Attached file 1:20190723_0020_EPCs-exo_1_size, raw data for the report.

Attached file 2:20190723_0020_EPCs-exo-2_1_size_11pos, raw data for the report.

Attached file 3:Video of individual particles, recorded video for the particle detection.
